# Physically plausible balloon dynamics via position-based constraints and geodesic-weighted forces

**DOI:** 10.1371/journal.pone.0348597

**Published:** 2026-05-13

**Authors:** Jong-Hyun Kim

**Affiliations:** College of Software and Convergence (Dept. of Artificial Intelligence, Design Technology), Graduate School of Electrical and Computer Engineering, Inha University, Michuhol-gu, Incheon‌‌, South Korea; University of Oxford, UNITED KINGDOM OF GREAT BRITAIN AND NORTHERN IRELAND

## Abstract

This paper presents a lightweight balloon‐dynamics method, built on the Position-Based Dynamics (PBD) framework, that reproduces real-time inflation–deflation–rotation as air is injected and released. Unlike volume/CFD approaches that require expensive fluid–structure coupling, our method avoids explicit fluid simulation by combining Bernoulli-derived reaction forces with PBD distance and volume constraints. Rotation is modeled as a global rigid-body motion (single rotation/quaternion update about the center of mass), while local shape changes are handled through constraint-based position correction—eschewing cluster-level or per-vertex local twisting. Geodesic-distance weighting of reaction forces and the separate treatment of translational and rotational components improve physical plausibility; minimal iterations and rigid-body rotation approximation preserve computational efficiency. Experiments on meshes with diverse geometries and mass distributions show consistent real-time performance on high-resolution models while capturing the characteristic balloon behaviors. The approach is well-suited for interactive applications such as games, VR/AR, and real-time physics-based content.

## Introduction

Balloons are more than just simple toys; they are objects that offer a wide variety of visual appeal and are used in fields such as advertising, exhibitions, and interactive content. In particular, rubber balloons exhibit complex and dramatic shape changes as air is injected, owing to their lightweight and flexible material properties, and the resulting physical dynamics are visually engaging. However, accurately reproducing the nonlinear inflation, rotation, and volume preservation characteristics of balloons remains one of the challenging tasks for existing simulation techniques.

Balloons have thin, elastic shell structures, and when large deformations are involved, it is difficult to simply apply thin plate or shell theories [1,2]. In general, such thin structures are numerically unstable to model directly as three-dimensional solids, and when the undeformed shape has curvature (e.g., balloons, leaves), even more complex analysis is required [3]. Although finite element methods (FEM) and fluid-simulation-based approaches have been used to address these issues, modeling with polyhedral meshes or considering internal fluid flow provides high accuracy at the expense of significant computational cost, making them unsuitable for real-time applications [4,5].

In the real world, balloon manufacturing processes are surprisingly simple, with the “dip molding” method—forming rubber over a basic mold shape—being the most common. This approach can produce balloons in a wide variety of shapes, but from a simulation perspective, there are still technical limitations in predicting and designing the deformation that will occur when air is injected. Balloon inflation is governed by a balance between elastic forces and internal pressure, which induces complex surface motion, and modeling this mathematically involves numerous constraints and intensive computation [6].

In this paper, we propose a new approach that can efficiently reproduce physical effects such as contraction, expansion, and rotation of a balloon’s surface caused by internal air, without the need for complex fluid dynamics simulation or fine mesh subdivision. The proposed method is based on Position-Based Dynamics (PBD), providing a framework that can reproduce surface deformations in a physically consistent manner without directly simulating airflow [7]. In particular, this technique enables simplified modeling of stretch-dominant deformation, volume preservation, and localized rotational effects that are characteristic of rubber balloons, and supports various initial shapes without mesh modification under the assumption of fixed thickness. Furthermore, experimental validation shows that the method maintains real-time performance even for high-resolution models, offering generality and efficiency for use in games, real-time content, interactive installations, educational simulations, and more.

### Problem statement‌‌

A rubber balloon is a representative elastic structure that exhibits nonlinear and complex behavior as air is injected and released. In particular, during air release, surface contraction, uneven internal/external pressure differences, and asymmetric shape changes cannot be explained by a simple inflation model, and they induce secondary motion effects such as rotation and lift-up. These phenomena occur as the escaping air generates thrust in a specific direction, and rotational inertia acts in conjunction with the displacement of the balloon’s center of mass. As a result, the balloon may rotate irregularly or soar upward, forming complex motion trajectories.

However, there are several challenges in accurately modeling such physical phenomena with existing simulation techniques:

1. **Nonlinear Large Deformation**: Initially thin and flexible, a balloon undergoes extreme variations in surface tension during air injection and release, with its overall shape and elasticity distribution changing over time. In particular, during air release, uneven contraction of the shape accelerates, producing unpredictable rotational motion.
2. **Difficulty in Modeling Thrust from Air Release**: As internal air escapes, subtle variations in airflow impose asymmetric pressures on parts of the surface, inducing rotational torque. Accurate computation of this requires complex fluid–structure interaction (FSI) analysis, which demands highly expensive computational resources.
3. **Dynamic Changes Over Time**: While air is being released, the balloon’s shape continuously changes, altering its center of mass, moment of inertia, and surface tension over time. All of these factors affect the balloon’s motion, and real-time computation requires highly precise time steps and a stable integrated simulation framework.
4. **Inefficiency in Real-Time Processing**: Accurately capturing all these factors necessitates high-resolution mesh subdivision, fine airflow analysis, and computation of elastic and reaction forces, which severely hinders real-time performance. In real-time applications such as games, XR environments, and physics-based virtual content, this computational load becomes a major bottleneck.

As a result, existing simulation approaches can only physically approximate the inflated state of a balloon, and have limitations in expressing complete dynamics including free flight, rotation, or lift-up caused by air release.

To address these limitations, this study proposes a simulation framework that can infer the rotation and lift-up motion of a balloon during air release using a simplified physics-based model, while leveraging PBD to achieve cost-efficient, real-time computation. This enables visually natural reproduction of the balloon’s dynamic motion, while providing a computational structure suitable for a variety of real-time content applications.

### How is the balloon effect typically created in the industry?

In 3D simulation-based content creation environments, a variety of techniques are employed to realistically reproduce the balloon effect, which includes inflation, deflation, rotation, and irregular motion caused by air release. In industrial contexts where balancing visual realism with simulation efficiency is critical, the following approaches are commonly adopted.

#### Mesh-based deformable simulation (FEM, mass-spring model).

The most traditional approach models the balloon’s surface using the Finite Element Method (FEM) or a Mass–Spring system, and simulates inflation and deflation effects through internal pressure conditions [8,9].

- The balloon is approximated as a thin and flexible shell, and the internal pressure is modeled as an external force acting in the normal direction.
- When air is injected, the distances between nodes increase, and the final shape is determined by the balance with elastic restoring forces.
- To simulate air release, elastic contraction and asymmetric force distribution caused by reduced pressure must be computed numerically.

However, FEM entails high computational cost, and in complex simulations, the lack of real-time performance makes it unsuitable for use in games or interactive environments.

#### Volume-preserving physics simulation.

In recent years, techniques that calculate elastic deformation while satisfying volume preservation conditions have become popular for simulating not only balloon inflation and deflation but also maintaining volume integrity [10–13].

- The PBD framework is a representative example.
- The balloon is represented as a thin shell composed of a triangular mesh, and in each frame, vertex positions are iteratively corrected to satisfy distance and volume constraints.
- This approach prevents the balloon from stretching beyond a certain volume while stably expressing natural inflation, rotation, and vibration effects.

PBD offers high computational efficiency and is integrated into real-time engines such as Unity and Unreal, making it widely used in games and AR/VR content.

#### Airflow coupling and force field approximation.

Free spinning motion and irregular ascent trajectories during air release are difficult to reproduce with simple inflation simulations alone; therefore, auxiliary models such as the following are often employed [14–16].

- Couple airflow (force field) or thrust (impulse) with a particle system or node-based structure to impart directional wind forces.
- Instead of using full Computational Fluid Dynamics (CFD), apply a virtual directional force to the rear of the balloon to induce rotational torque or lift acceleration.
- Such forces are dynamically adjusted in each frame according to the balloon’s asymmetric surface, normal vectors, and outflow rate, enabling visual effects similar to real-world physical behavior.

#### Hybrid approach with animation control.

To replace complex fluid–structure interaction (FSI) simulations, hybrid methods that combine physics-based simulation with keyframe animation are also commonly used.

- Inflation, oscillation, and rotation are handled with physics-based calculations.
- Highly unpredictable or strongly nonlinear rotational trajectories are supplemented with control curves or physically motivated animation tracks.
- This approach maintains realism while managing computational cost, making it well-suited for production pipelines.

#### Summary.

These various approaches are selected according to the intended purpose of the content (real-time or offline rendering), required accuracy, and system performance (see Table 1). Recently, due to industrial demands for efficiency and scalability, PBD-based simulation techniques have been gaining mainstream adoption [13]. However, existing PBD applications are largely limited to simple structural deformations and have not sufficiently addressed balloon simulations that incorporate asymmetric rotation, nonlinear contraction, and complex dynamic changes over time during air release.

**Table 1:** Comparison of different approaches for simulating balloon effects.

Approach	Characteristics	Advantages	Limitations
FEM, Mass-Spring	Structurally precise	Accurate stress/deformation analysis	High computational cost, not suitable for real-time use
PBD (Volume-preserving)	Lightweight structural dynamics	Real-time performance, numerical stability	Limited accuracy in aerodynamic behavior
Force Field Coupling	Rotation and lift motion expression	Indirect modeling of air release dynamics	Lacks accurate airflow calculation
Hybrid (Physics and Animation)	Realistic motion control	High controllability	Difficult to fully automate

To address these limitations, this paper proposes a lightweight PBD-based simulation method that can represent not only rotation and volume changes but also complex dynamic phenomena arising from elasticity without simulating internal airflow. The proposed method effectively reproduces the nonlinear behavior characteristic of balloons while minimizing computational cost for real-time applications, making it suitable for integration into games, interactive content, XR environments, and other real-time physics-based systems.

## Related work

The simulation of deformable objects has long been a central research topic in computer graphics and animation. Early work began with the finite difference-based physical simulation method proposed by Terzopoulos et al. [17], followed by various approaches such as the mass–spring system [18,19], the boundary element method [20], and the finite element method (FEM) [21]. These methodologies have contributed to modeling the elastic and dynamic behavior of a wide range of structures, and the overall developments in this field have been reviewed in survey papers [22].

Subsequently, Wu et al. proposed a method for accurately computing the behavior of nonlinear membranes using FEM [23], while Volino et al. developed a cloth simulation model based on continuum mechanics that reproduced deformation patterns close to those of real textile materials [24]. Based on the observation that large deformations are often induced by rotation, the corotated finite element method (Corotated FEM) was introduced [25,26], achieving a balance between accuracy and efficiency. However, this method had limitations in reproducing stretch-dominant behavior. To address this, McAdams et al. utilized hexahedral elements to efficiently simulate deformations of soft tissues such as skin, though their approach was not applicable to generalized triangular mesh structures [27].

Simulating thin shells is considered a numerically challenging problem due to their degenerate nature, where one dimension effectively vanishes because the thickness is extremely small compared to the span. This topic has been actively studied not only in physics-based simulation but also in CAD and graphics communities, with various numerical approaches proposed. Arnold analyzed the numerical stability issues of thin shells [28], and Cirak et al. integrated a subdivision basis into FEM based on Kirchhoff–Love theory to improve accuracy [29]. Expanding this to the graphics domain, Green et al. enhanced computational efficiency through multilevel solvers [30], and Grinspun et al. presented a more compact and general discrete shell framework [31]. They later proposed a simplified computational structure capable of real-time processing for shell structures that undergo significant bending, such as paper, cloth, and hats [2].

Meanwhile, the simulation of thin-shell structures with internal pressure has also been actively investigated in related applications. Bonet et al. modeled pressurized shells using techniques from cloth simulation; however, their reliance on 2D material points made it difficult to handle complex geometries [1]. To overcome this limitation, Skouras et al. modified the definition of the deformation gradient to ensure volume preservation for each element during deformation, while removing the requirement for material points [6]. Nonetheless, this approach can make the numerical system excessively complex, leading to ill-conditioned problems and stability limitations.

Various simulation techniques have attempted to address the structural characteristics of thin shells and the physical constraints of elastic deformation and volume preservation through a multitude of numerical models and element-based approaches. However, many of these studies relied on fixed mesh structures or high-cost computation to ensure accuracy, and few have incorporated complex dynamic phenomena such as rotation, asymmetric contraction, and aerodynamic reaction forces.

Moreover, most methods have been designed for static or limited deformation conditions, making it difficult to represent nonlinear rotation and surface motion in real time under dynamic internal pressure changes caused by air injection and release. This limitation becomes even more apparent in structures like rubber balloons, where strong interactions occur between the internal fluid and the elastic shell.

Ultimately, developing a lightweight, physics-based approach that can stably represent complex surface rotation and asymmetric deformation induced by the air inside a balloon in real time remains an open challenge, and addressing this technical gap forms the starting point of this study.

## Preliminaries

### Position-based dynamics

Position-Based Dynamics (PBD) is a technique for efficiently modeling deformable objects such as in cloth simulation. The core idea of PBD is to first estimate predicted positions under external forces using Euler’s method, and then iteratively apply constraint equations, based on Hooke’s law, to correct positions so that internal forces are satisfied. The overall solver consists of two main steps: predicted position calculation due to external forces, and position correction to satisfy the constraints. Among these, the predicted position is calculated using Euler integration as follows.


$$\begin{array}{cc} v_{i} & =v_{i}+\Delta tw_{i}f_{\mathrm{ext}}(x_{i})\\ p_{i} & =x_{i}+\Delta tv_{i} \end{array}$$ (1)

Here, *v* is the velocity, *w* is the inverse mass, *f*_ext_ is the external force, *x* is the previous position, *p* is the predicted position, and *i* denotes the index of the vertex. To correct *p* so that it satisfies the constraints, the Gauss–Seidel method is used. Since the equations for each constraint are nonlinear, the solution is found numerically by iteratively reducing the error. Finally, the position and velocity at the next time step are computed using:


$$\begin{array}{c} \begin{array}{c} \begin{array}{cc} v_{i} & =\frac{p_{i}-x_{i}}{\Delta t}\\ x_{i} & =p_{i} \end{array} \end{array} \end{array}$$ (2)

The key in PBD is to project points onto the constraint manifold to find the optimal solution. Since these constraints are due to internal forces such as those described by Hooke’s law, rather than external forces, momentum must be conserved. To this end, in the following equation, the gradient of *C*, ∇C, is assumed to be rigid, and the optimal solution for the constraint is found:


$$C(p+\Delta p)\approx C(p)+\nabla_{p}C(p)\cdot \Delta p=0$$ (3)

To assume ∇C as rigid, the direction of Δp is assigned to ∇C. Expanding the equation allows computation of the position change Δp that each vertex must undergo. For an individual vertex, Equation 3 can be expressed as follows:


$$\begin{array}{c} \begin{array}{c} \begin{array}{cc} \Delta p_{i} & =-sw_{i}\nabla_{p_{i}}C(p_{1},\ldots ,p_{n})\\ s & =\frac{C(p_{1},\ldots ,p_{n})}{\sum_{j}w_{j}{\left|\nabla_{p_{j}}C(p_{1},\ldots ,p_{n})\right|}^{2}} \end{array} \end{array} \end{array}$$ (4)

### Position-based constraints

Position-Based Constraints are a core concept of the Position-Based Dynamics (PBD) framework. Instead of computing motion from forces or accelerations, this approach directly adjusts the *positions* of vertices to satisfy given physical or geometric constraints through iterative projection. This position-level formulation significantly reduces numerical instability and enables real-time computation, making it suitable for interactive simulations such as deformable objects or soft-body dynamics.

In PBD, the simulation first predicts vertex positions under external forces (e.g., gravity, reaction force), and then iteratively corrects those positions so that the defined constraints (e.g., distance, volume) are satisfied. The correction step projects each vertex onto the constraint manifold, ensuring that the deformation remains physically plausible.

In this study, two types of Position-Based Constraints are employed:

- Length Constraint: Preserves the initial edge lengths between adjacent vertices to maintain structural elasticity.
- Volume Constraint: Maintains the initial enclosed volume of the surface mesh to control the inflation and deflation behavior of the balloon.

These constraints are repeatedly applied during the iterative correction stage of the PBD solver, allowing the system to maintain physically consistent deformation throughout the simulation. Consequently, in this work, the term *Position-Based Constraints* refers to the position-driven physical constraint structure that enables stable and real-time reproduction of the balloon’s complex behaviors such as inflation, deflation, and rotation.

## Proposed method

### Constraint

The constraints used in this study can be categorized into length constraints and volume constraints. The length constraint preserves the initial length, similar to a mass–spring system, and is computed as follows:


$$C(p_{1},p_{2})=\left|p_{1}-p_{2}\right|-d$$ (5)

By substituting the above equation into Equation 4, we obtain the result shown in Equation 6. When this is applied to the distance constraint, Equation 3 can be satisfied.


$$\begin{array}{c} \begin{array}{c} \begin{array}{cc} \Delta p_{1} & =-\frac{w_{1}}{w_{1}+w_{2}}\left(\left|p_{1}-p_{2}\right|-d\right)\frac{p_{1}-p_{2}}{\left|p_{1}-p_{2}\right|}\\ \Delta p_{2} & =+\frac{w_{2}}{w_{1}+w_{2}}\left(\left|p_{1}-p_{2}\right|-d\right)\frac{p_{1}-p_{2}}{\left|p_{1}-p_{2}\right|} \end{array} \end{array} \end{array}$$ (6)

**Fig 1:**
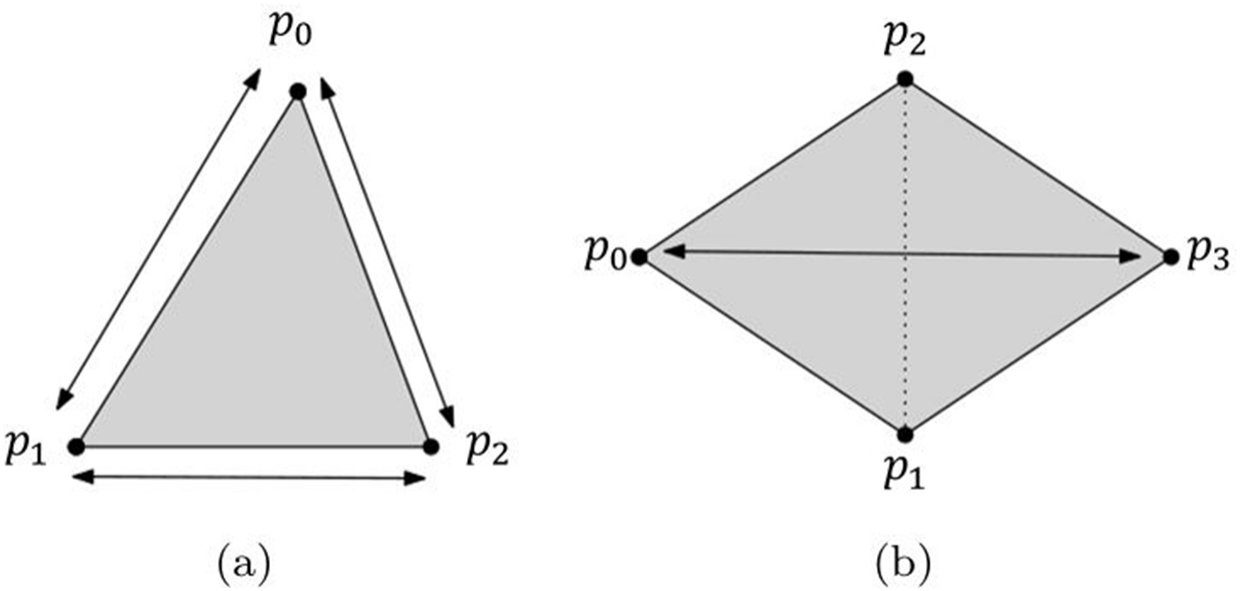
Generation of structural and bending springs.

In this work, the length constraint is used to generate *structural springs*, which connect vertices, and *bend springs*, which connect crossover edges. Since a triangular mesh is used, the structural springs connect vertices according to the triangle’s vertex indices [0,1,2] (see Fig 1a). The bend springs are created by connecting two common neighbor vertices, excluding the selected vertex, so that two vertices completely opposite each other across the shared edge of two triangles are connected (see Fig 1b).

Next, the volume constraint, similar to the length constraint, is computed by subtracting the initial volume from the current volume (see Equation 7). The gradient for the volume constraint is calculated using Equation 8.


$$C(p_{1},\ldots ,p_{n})=\left(\sum_{i=1}^{n_{\Delta s}}\left(p_{t_{1}^{i}}\times p_{t_{2}^{i}}\right)\cdot p_{t_{3}^{i}}\right)-V_{0}$$ (7)

$$\begin{array}{c} \begin{array}{c} \begin{array}{cc} \nabla_{p_{i}}C= & \sum_{j:t_{1}^{j}=i}\left(p_{t_{2}^{j}}\times p_{t_{3}^{j}}\right)+\sum_{j:t_{2}^{j}=i}\left(p_{t_{3}^{j}}\times p_{t_{1}^{j}}\right)+\\ \sum_{j:t_{3}^{j}=i}\left(p_{t_{1}^{j}}\times p_{t_{2}^{j}}\right) \end{array} \end{array} \end{array}$$ (8)

**Fig 2:**
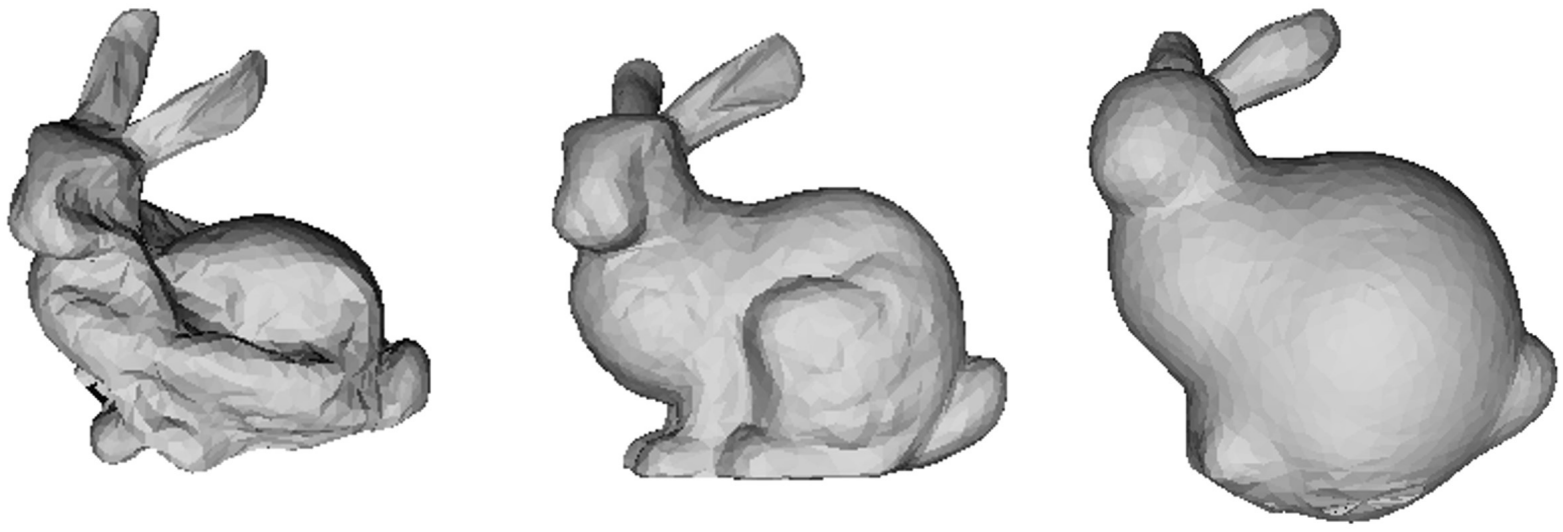
Simulation of the volume constraint.

Substituting the above equations into Equation 4 yields $\Delta p_{i}$. Applying this allows the simulation to produce different results depending on the initial volume. Fig 2 clearly demonstrates the surface contraction and expansion of the Stanford Bunny model due to the volume constraint.

### Balloon effects

In this study, the initial volume *V*_0_ used in the volume constraint is leveraged to reproduce the balloon effect. When air is injected, *V*_0_ is increased; when air is released, *V*_0_ is decreased. This naturally produces surface deformations in which the object’s volume expands or contracts. The change in balloon volume during inflation and deflation is due to variations in the internal pressure. When the internal pressure exceeds atmospheric pressure, the volume increases; when it falls below atmospheric pressure, the volume decreases. Therefore, the internal pressure *PV* of the balloon is calculated using the Ideal Gas Law in Equation 9, and air release is stopped when the pressure equals atmospheric pressure.


PV=nRT (9)

Here, *n* is the number of moles of gas, and *R* and *T* are the gas constant and temperature, respectively. In this study, atmospheric pressure is set to 101,325 Pa, the temperature to 293.15 K, and the gas is assumed to be helium. The number of moles of gas is approximated based on the additional volume inside the balloon.

#### External force.

Four external forces are considered in this study: gravity, buoyancy, air resistance, and reaction force. Gravity *g* is set to its standard value of 9.8 m/s^2^, and buoyancy *F*_buoy_ is calculated using Equation 10. If the buoyancy exceeds gravity, the balloon will rise. The densities are set as follows: $\rho_{ext}$ is the density of the ambient air, and $\rho_{inter}$ is the density of the gas (helium) inside the balloon.


$$F_{buoy}\;=\;-\;g\;V\;(\rho_{ext}-\rho_{inter})$$ (10)

Here, *V* denotes the volume of the balloon. Air resistance *F*_drag_ is computed using Equation 11. To weight the resistance according to the direction of motion, the velocity vector is dotted with the surface normal vector. The drag coefficient *C*_*d*_ is set to 0.5.


$$F_{\text{drag}}=-\frac{1}{2}\rho v^{2}AC_{d}\frac{\left(v\cdot n\right)}{\left\|v\right\|}$$ (11)

Here, ρ is the fluid density, *v* is the velocity vector, *A* is the reference (projected) area, *C*_*d*_ is the drag coefficient, and *n* is the surface normal vector.

Finally, the effect of air escaping from the balloon is modeled based on reaction force. Before computing the reaction force, Bernoulli’s equation is considered. Air inside the balloon is at high pressure, and as it escapes through a narrow opening, the pressure difference accelerates the flow. The relationship between fluid velocity and pressure can be described using Bernoulli’s equation, given in Equation 12.


$$P+\frac{1}{2}\rho v^{2}+\rho gh=\text{constant}$$ (12)

Here, *P* is the pressure, ρ is the air density, *v* is the flow velocity, and *h* is the height. Applying this equation between the inside of the balloon (upstream of the outlet) and the outside (downstream of the outlet) shows that a greater pressure difference results in a higher outlet velocity. According to Newton’s third law of motion, the change in momentum of the rapidly escaping air produces a force in the opposite direction—this is the reaction force—which propels or rotates the balloon. When the balloon’s opening is offset from the center, asymmetric forces and torques arise, inducing rotation. In this study, the outlet velocity and pressure distribution are first estimated from Bernoulli’s equation, then converted into momentum change rate to obtain the corresponding force and torque.

In Equation 12, the second term on the left-hand side represents kinetic energy and the third term potential energy. Initially, the kinetic energy of the escaping fluid is zero, so the constant on the right-hand side can be determined at initialization. This constant remains fixed, allowing the velocity *v* of the escaping fluid to be computed. Multiplying this velocity by the normal vector at the outlet vertex gives the applied force of the escaping fluid, and the reaction force is applied in the opposite direction to model the air release effect.

In PBD, deformation and motion are not directly integrated using force-based methods; instead, the simulation iteratively corrects positions. However, external forces can still be indirectly incorporated by updating velocities, which is crucial for achieving physically plausible motion. In this study, the outlet velocity *v* and fluid density ρ obtained from Bernoulli’s equation are used to calculate the impulse, which is then converted into a reaction force applied to the balloon’s surface particles.


$$F_{\text{reaction}}=\dot{m}\cdot v=(\rho \cdot A\cdot v)\cdot v$$ (13)

Here, *A* is the outlet cross-sectional area, and *m* is the mass flow rate per unit time. This force generates translational acceleration at the balloon’s center of mass and rotational torque via the cross product with the outlet position vector.

During the PBD update process, the reaction force is incorporated into the velocity update step at each simulation iteration as shown in Equation 14:


$$v_{i}\leftarrow v_{i}+\frac{F_{\text{reaction}}}{m_{i}}\cdot \Delta t$$ (14)

Through this procedure, the model does not simply animate air release, but physically incorporates reaction forces based on fluid dynamics into the PBD simulation. As a result, the balloon’s forward motion, rotation, and oscillation—caused by variations in air release speed, direction, and outlet position—are reproduced naturally.

**Fig 3:**
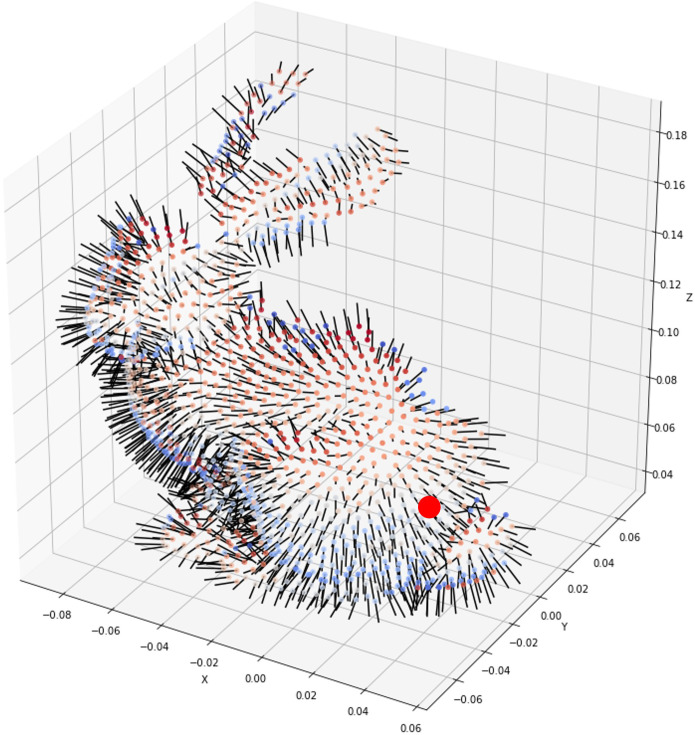
Candidate positions and directions for air release. The red point marks a randomly selected outlet‌‌ location.

Fig 3 illustrates an example of candidate positions and directions for air release. The red point indicates a randomly designated air outlet location, and the surface normal vector at this point determines the primary direction of air flow. These candidate position and direction data serve as the initial conditions for reaction force calculation. The translational and rotational behavior of the balloon varies depending on the outlet’s distance from the center of mass and the orientation of its normal vector. In particular, candidate configurations such as those in Fig 3 are used to experimentally compare various position–direction combinations and to analyze the resulting asymmetric motion and torque variations during air release.

#### Torque.

When air is released from a balloon, the resulting motion includes not only translational movement but also rotational motion. However, because a balloon is a deformable body, computing rotation typically requires partitioning the space into clusters to extract local rotations for each region, or calculating rotational components individually for all vertices. Such approaches significantly increase computational load due to repeated displacement tracking and coordinate transformations, making them inefficient for real-time simulations.

In this work, the goal is to implement the balloon effect in real time. Therefore, rotation is approximated by treating the balloon as a rigid body and unifying it into a single rotation matrix. In practice, as the internal air pressure increases, the balloon surface bulges in the direction of its normal vector. This means that, unlike general deformable bodies, local rotations are not overly complex. As a result, applying a single rotation matrix is possible without compromising visual quality, achieving both efficiency and realism in visual simulation.

The torque τ is calculated as the cross product of the point of application *p* and the force vector *F* (see Equation 15). Under the rigid-body approximation, the point of application is defined in the local coordinate system relative to the center of mass.


τ=p×F (15)

Analogous to calculating linear acceleration in translational motion (see Equation 1), rotational motion is computed using the applied torque τ and the inverse inertia tensor *I*^−1^ to obtain the angular acceleration $\dot{\omega }$ (see Equation 16). The angular acceleration is then integrated over time to update the angular velocity and orientation, which are subsequently expressed as either a rotation matrix or a quaternion and applied to all vertices in the local coordinate system to realize the overall rotation (see Equations 17, 18). Here, ω denotes the angular velocity represented in quaternion form.


$$\dot{\omega }=I^{-1}\;\tau$$ (16)

$$\omega =\omega +\Delta t\;\dot{\omega }$$ (17)

$$\theta =\theta +\frac{\Delta t}{2}\;\omega \;\theta$$ (18)

The inverse inertia tensor *I*^−1^ is first computed in the local coordinate system (Equations 19, 20) and is then transformed into the world coordinate system for use during the simulation. For rigid bodies, the inverse inertia tensor is computed once during preprocessing, but for deformable bodies, it must be updated at every simulation step since the center of mass and shape change over time.


$$I_{xy}=\sum_{i=1}^{n}m_{i}\;(p_{i}\cdot x)(p_{i}\cdot y)$$ (19)

$$I=[\begin{array}{ccc} I_{x} & -I_{xy} & -I_{xz}\\ -I_{xy} & I_{y} & -I_{yz}\\ -I_{xz} & -I_{yz} & I_{z} \end{array}]$$ (20)

The following optimization strategies were applied in this study:

- Approximate the rotation of the deformable body as a single rigid-body rotation, eliminating the need for cluster-based rotation computations.
- Minimize real-time updates of the inverse inertia tensor by reusing its value from the previous step in regions with minimal shape change.
- Use quaternion-based integration for rotation to prevent accumulated errors that can occur with matrix-based integration.
- Integrate translational and rotational motion processing by structuring the PBD simulation loop as a unified stage: velocity update → position prediction → rotation application.

With these optimizations, the rotational motion of the balloon can be simulated in real time with both physical plausibility and computational efficiency.

## Solver extensions

### Influence of air release location on translational and rotational dynamics

This section analyzes how the air release point affects the balloon’s translational and rotational motion. The reaction force is computed based on the outlet cross-sectional area *A*, the outlet velocity *v*, and the normal vector at that location, and this force is then transferred to the balloon’s center of mass and all surface particles. In summary, the other vertices do not have fixed weights; rather, their weights are determined dynamically according to how the reaction force is distributed to all particles during the velocity update step in the PBD external force calculation. Specifically:

1. Effect of the Outlet Normal Direction
  - The direction of the escaping fluid from the outlet is defined by the outlet’s normal vector.
  - The degree to which the outlet position and each vertex’s relative position align with the normal vector influences how the force is transmitted.
2. Mass-Based Weighting
  - Each vertex *i* receives a reaction force proportional to its mass *m*_*i*_.
  - As shown in Equation 14, smaller *m*_*i*_ results in a larger change in velocity during the PBD update.
3. Translational vs. Rotational Distribution
  - The translational component is applied evenly to all vertices relative to the center of mass.
  - The rotational component is weighted according to the torque calculation (see Equation 15), based on each vertex’s position vector relative to the center of mass. Vertices farther from the center of mass experience larger changes in angular velocity.

Thus, the weights for the other vertices are dynamically determined by the combination of “distance from the center of mass,” “mass,” and “normal direction of the air release point.”

One might ask whether changing the air release point location has little impact. The answer is that the air release point is a highly significant parameter, and changing it will alter the results for the following reasons:

1. Change in Reaction Force Direction
  - The reaction force *F*_reaction_ is calculated from Equation 13, where the direction of *v* is determined by the outlet’s normal vector.
  - When the location changes, the normal vector direction changes, altering the overall force direction.
  - This directly affects both the direction and magnitude of translational motion.
2. Change in Torque Magnitude and Direction
  - In the torque calculation (see Equation 15), *p* is the outlet position vector relative to the center of mass.
  - The farther the outlet is from the center of mass, the greater the torque; the closer it is to the same axis as the center of mass, the smaller the torque.
  - Therefore, changing the outlet position changes both the angular velocity and the axis of rotation.
3. Change in Per-Vertex Force Distribution
  - The reaction force is divided into translational and rotational components and applied to each vertex.
  - Moving the outlet alters the distribution of the rotational component, causing certain areas to oscillate more or changing the overall rotational motion.

In short, changing the outlet location alters the translational motion direction, the magnitude and direction of rotation, and the surface deformation patterns. Asymmetrical locations amplify rotational effects, while positions closer to the center of mass produce weaker rotation and stronger linear movement.

However, the approach described above does not explicitly take into account the distance between the air release point and each vertex. In the reaction force calculation (see Equation 13), *v* is aligned with the outlet’s normal vector, and its magnitude is obtained from Bernoulli’s equation. When this force is uniformly applied to the PBD external force velocity update, all vertices are affected by the same direction vector. In other words, there is no attenuation (weight falloff) based on positional distance or directional difference. For rotational effects as well, the torque calculation τ=p×F only considers the outlet position *p* relative to the center of mass, and does not directly account for each vertex’s distance in this step. Consequently, the air release vector determines only the “force direction” and “magnitude,” meaning that vertices near and far from the outlet receive the same magnitude of translational component. Thus, the physical phenomenon in which “areas closer to the outlet are more strongly affected” is not reproduced.

### Geodesic-weighted reaction force distribution

As analyzed in the previous section, the conventional approach does not directly account for the distance between the air release point and each vertex, and therefore cannot fully reproduce the physical phenomenon in which regions closer to the outlet experience stronger effects. To address this limitation, this section proposes a method for distributing the reaction force based on the geodesic distance computed along the mesh surface, together with the formulation and implementation details.

The weight for vertex *i* is defined as follows:


$$w_{i}=\exp\;[-(\frac{d_{g}(x_{i},x_{\text{exit}})}{\sigma }{)}^{p}],\;{\hat{w}}_{i}=\frac{w_{i}}{\sum_{j}w_{j}}$$ (21)

Here, $d_{g}(x_{i},x_{\text{exit}})$ is the geodesic distance between vertex *x*_*i*_ and outlet *x*_exit_, σ is a scale parameter controlling the radius of influence, and *p* is an exponent controlling the attenuation curve shape. In addition, the directional alignment between the outlet direction and the vertex normal vector *n*_*i*_ is incorporated via their dot product:


$$\alpha_{i}=\max(0,\;n_{i}\cdot {\hat{v}}_{\text{exit}}),\;{\tilde{w}}_{i}=\frac{\alpha_{i}\;{\hat{w}}_{i}}{\sum_{j}\alpha_{j}\;{\hat{w}}_{j}}$$ (22)

Finally, the reaction force applied to each vertex is computed as:


$$F_{i}={\tilde{w}}_{i}\cdot F_{\text{reaction}},\;v_{i}\leftarrow v_{i}+\frac{F_{i}}{m_{i}}\cdot \Delta t$$ (23)

where *m*_*i*_ is the vertex mass and Δt is the simulation time step.

The geodesic distance can be computed using a single-source shortest path algorithm (Dijkstra) on the mesh graph, or via the heat method [32] for a smoother approximation. To reduce computational cost, distances are computed only within a radius *R* from the outlet, and distances from the previous frame are reused when geometry changes between frames are small.

This approach offers the following advantages:

- Improved physical plausibility: Regions closer on the surface receive larger forces, reproducing localized deformations observed in actual air release.
- Enhanced asymmetric deformation: Preserves local rotation and asymmetric contraction effects dependent on outlet location.
- Stability and controllability: Parameters σ, *p*, and *R* allow control over the influence range and attenuation rate.

## Experimental results

This paper proposes a real-time method to represent *inflation–deflation–rotation* behavior due to air injection/release by combining PBD constraints (distance, volume) with external forces (gravity, buoyancy, air drag, reaction force), without directly simulating internal/external fluids. In particular, the reaction force is computed from the outlet normal direction and velocity, and delivered to each vertex in a physically plausible magnitude using the proposed distribution strategies (mass-based, translational/rotational decomposition, optionally geodesic-weighted). As a result, changing the air release point naturally alters the translation direction, rotation axis and angular velocity, and surface wrinkle patterns.

In the experiments, the *Stanford Bunny* model composed of a triangular shell was used as a closed (balloon-like) structure. Volume constraints control inflation/deflation by maintaining or reducing the initial volume *V*_0_, and the external forces include gravity, buoyancy, air drag, and the reaction force computed from the outlet cross-sectional area *A* and velocity *v*. Rotation is computed from the torque τ=p×F relative to the center of mass, integrated into angular velocity using the inverse inertia tensor (quaternion-based), and in each frame, position correction (constraint projection) and velocity updates are performed.

**Fig 4:**
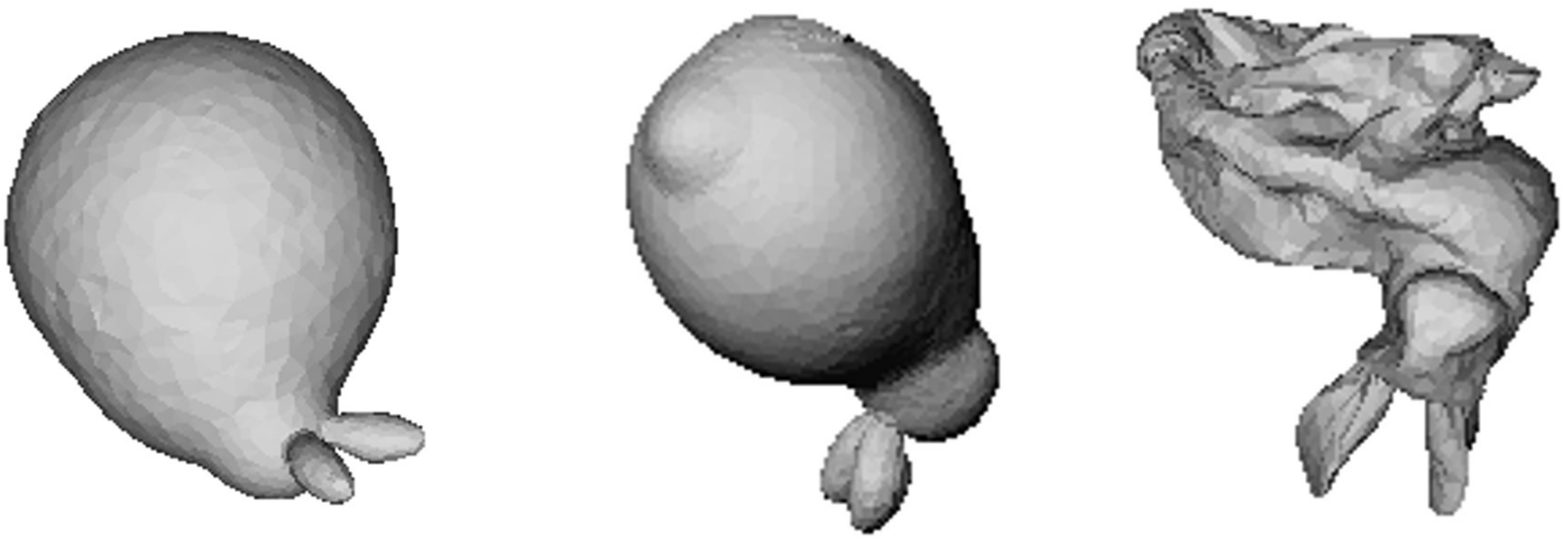
Physical deformation and rotation of the balloon in response to air release, simulated using our method.

Fig 4 shows the sequential process of *inflation–deflation–collapse* with air injection/release:

- Left (Inflated): The volume constraint maintains *V*_0_, keeping the surface uniformly inflated. The balance between structural and bend springs preserves a smooth exterior without excessive stretching.
- Middle (Early Deflation with Asymmetry): The reaction force from the outlet induces torque relative to the center of mass, initiating *rotation* along with subtle asymmetric contraction. Areas aligned with the outlet normal compress first, while distant regions deform later due to inertia.
- Right (Late Deflation/Collapse): As internal pressure decreases sufficiently, the shell wrinkles and collapses under gravity and air drag. Constraint-based position correction prevents triangle intersections, and bend springs control excessive folding, allowing stable wrinkle patterns without visual artifacts.

**Fig 5:**
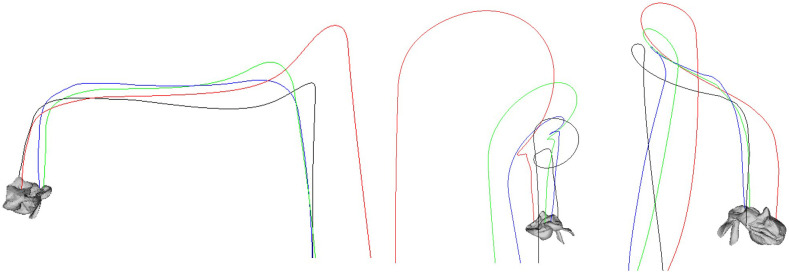
Balloon-induced rotation under our PBD framework. Four surface points are tracked in world coordinates (color-coded trajectories) during air release. Curved paths and tightening spirals indicate increasing torque and angular velocity over time.

Fig 5 visualizes the trajectories of four reference surface vertices, distinguished by color (e.g., red/blue/green/black), to qualitatively analyze the rotation occurring during air release. In the initial phase, the reaction force primarily acts as a translational component, producing gently curved trajectories. As air release continues, torque generated from the outlet position relative to the center of mass increases, and the trajectories exhibit more curvature and pronounced spiral motion. The gradually shrinking spiral radius and shorter rotation period indicate an increase in angular velocity. Furthermore, each vertex follows a different trajectory depending on its distance from the center of mass and alignment with the surface normal, as the decomposition of the reaction force (translation/rotation) combines with directional alignment (normal dot product) to induce asymmetric deformation. This visualization clearly shows how increased air release amplifies rotation torque, resulting in stronger spiral trajectories around the rotation axis. When geodesic weighting is applied, curvature near the outlet vertices becomes more pronounced, further enhancing localized rotational responses.

**Fig 6:**
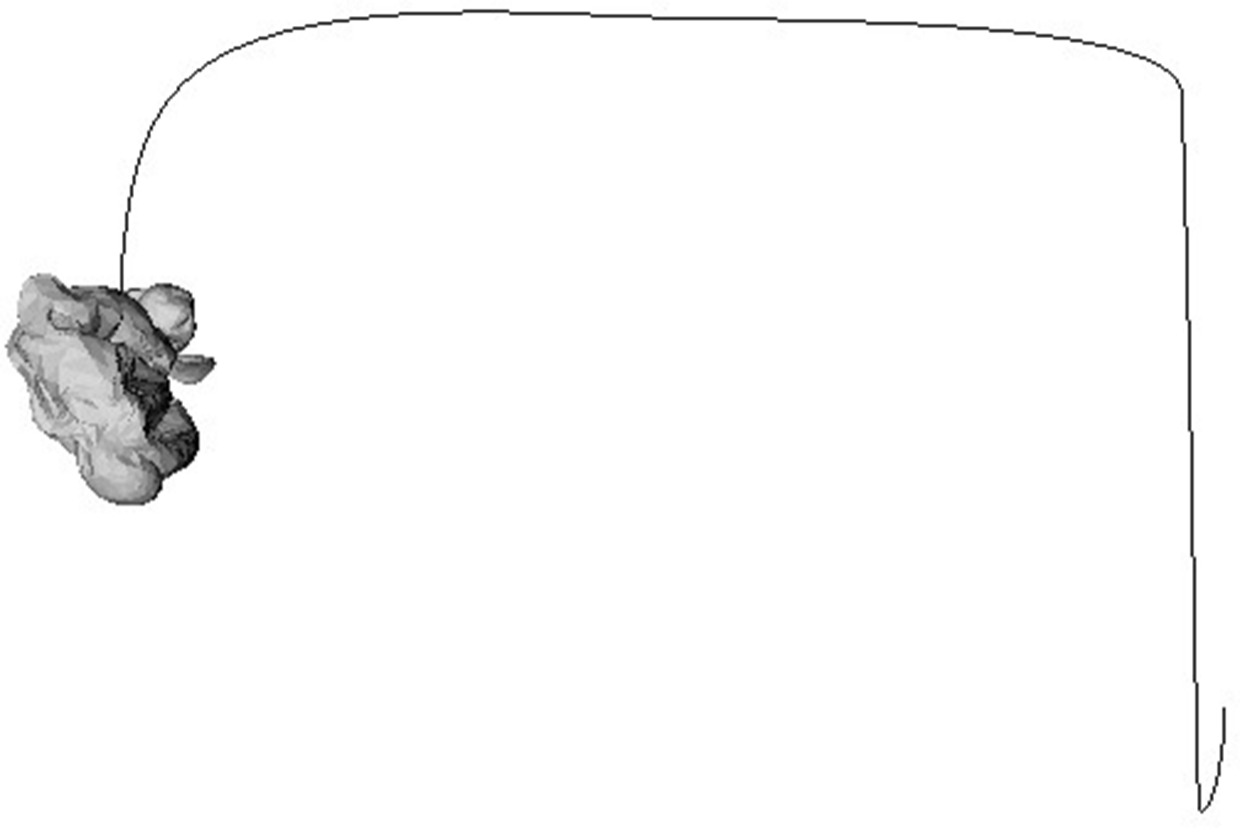
Inflation of a balloon followed by sudden air release. The trajectory shows the transition from buoyancy-dominated ascent to reaction-force-driven motion, followed by decay as internal pressure drops.

Fig 6 visualizes the global trajectory when air is first injected and then abruptly released. While the internal pressure is maintained, the volume constraint keeps the balloon inflated and buoyancy exceeds gravity (*F*_buoy_ > *mg*), resulting in a gentle *ascent*. At the instant of release, the reaction force *F*_reaction_ aligned with the outlet normal generates lateral acceleration and a torque τ=p×F, bending the path into a large *arc* and initiating rotation. As the internal pressure decays, both the reaction force and buoyancy decrease, leading to a gradual reduction of linear and angular velocities and a final *settling* phase at the end of the trajectory.

**Fig 7:**
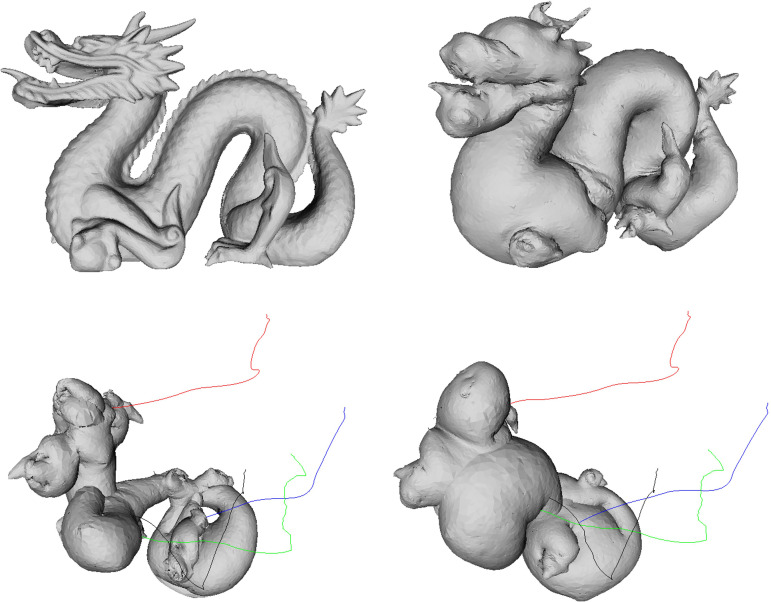
Balloon-like dynamics of the Stanford Dragon model under our PBD-based simulation. The sequence captures complex deformation and motion patterns arising from the model’s elongated, articulated geometry, including asymmetric translation during deflation and coupled rotation–ascent during reinflation.

Fig 7 shows the result of applying the proposed PBD-based simulation to the Stanford Dragon model, demonstrating that constraint-based inflation–deflation behavior is stably reproduced even in models with complex geometry. The dragon model includes an elongated body, curved tail, and sharp protrusions, making its mass center and surface element distribution more asymmetric compared to a simple spherical shell. In the experiment, when the model was inflated and then deflated, differences in mass distribution between the tail and head caused the central axis to tilt, producing asymmetric translational motion first. Subsequently, uneven application of reaction force on parts of the surface induced irregular rotation along the head–tail axis.

During reinflation, restoration of the volume constraint pushed the tail backward, while the relatively lighter head rose, producing coupled rotation–ascent behavior. The trajectory visualization (color-coded lines) shows that this motion is not confined to a single plane but unfolds in three dimensions according to the model’s complex shape. In particular, regions with high curvature and protrusions exhibited stronger surface deformation and rotational response during reinflation. This result demonstrates that the proposed method operates stably even on high-resolution, complex shapes and can reproduce rich motion patterns arising from asymmetric mass distribution and complex surface geometry.

**Fig 8:**
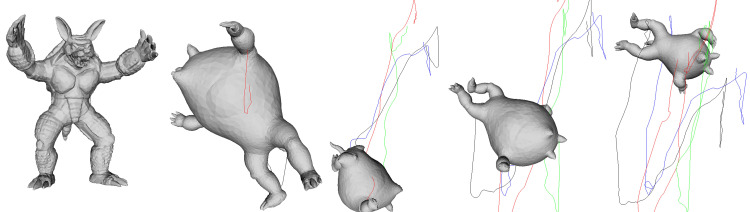
Balloon-like motion of the Armadillo model in our PBD simulation. The sequence shows how the model’s irregular limb and torso geometry cause distinctive balance shifts, complex rotational responses, and trajectory deviations during alternating inflation–deflation cycles.

Fig 8 presents the simulation results for the Armadillo model, which has complex limbs and an asymmetrical torso. Unlike a simple shell with uniform mass distribution, this model combines protruding parts such as arms, legs, and head with a large torso, resulting in significant shifts in the center of mass during inflation and deflation. In the inflated state, buoyancy and initial velocity allow the model to ascend in a relatively stable posture. However, upon switching to deflation, the asymmetric mass distribution of the long arms and legs creates imbalance in the rotation axis, initiating irregular rotation. In particular, when one limb is oriented downward, the distribution of air drag changes, causing abrupt deviations in both rotational speed and trajectory.

During reinflation, as internal pressure recovers, the total volume expands again, and changes in the relative positions of the limbs and torso momentarily realign the rotation axis. Consequently, the trajectory follows a pattern of continuous deviation–correction–ascent rather than straight ascent, and the color-coded trajectory lines show that this motion is not limited to a single axis but evolves in a complex manner in three-dimensional space. This experiment demonstrates that the proposed method can reliably represent chained behaviors of inflation–deflation–rotation–ascent even for challenging meshes with numerous protrusions and nonuniform mass distribution.

**Fig 9:**
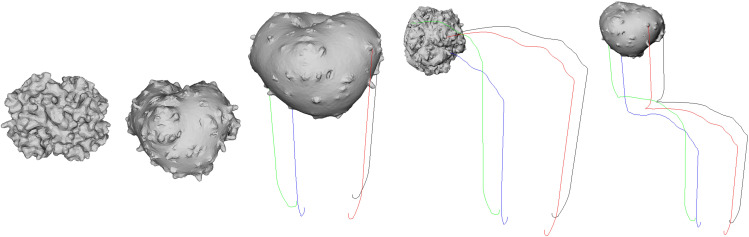
Balloon-like behavior of a sharp-featured model under alternating inflation–deflation. The sequence illustrates how surface protrusions and outlet orientation influence lateral translation and rotational dynamics, producing non-vertical trajectories.

Fig 9 shows the result of applying the proposed method to a model with numerous sharp protrusions and irregular surface geometry. Such shapes have a nonuniform distribution of surface normal directions, which tends to concentrate reaction force in specific directions during air release. In the inflated state, the volume constraint maintains stability, and buoyant ascent occurs smoothly. However, when deflation begins, the reaction force vector changes according to the outlet position and orientation, which can induce lateral translation instead of downward motion. In this experiment, the outlet was located at the upper left of the model, with its normal vector tilted leftward, so at the onset of deflation the model experienced leftward-biased translation along with asymmetric torque, initiating rotation.

During reinflation, as internal pressure recovers, the rotation axis realigns and the ascent component regains dominance, moving the model upward. However, residual rotational inertia results in a curved, three-dimensional trajectory rather than purely vertical ascent, as clearly seen in the color-coded trajectory lines. This result indicates that the direction of motion during air release is not restricted to downward movement; rather, the combination of outlet location, normal direction, and surface geometry strongly influences both translational and rotational behavior. This confirms that the proposed method can naturally reproduce a wide variety of motion patterns even for meshes with high surface complexity.

**Fig 10:**
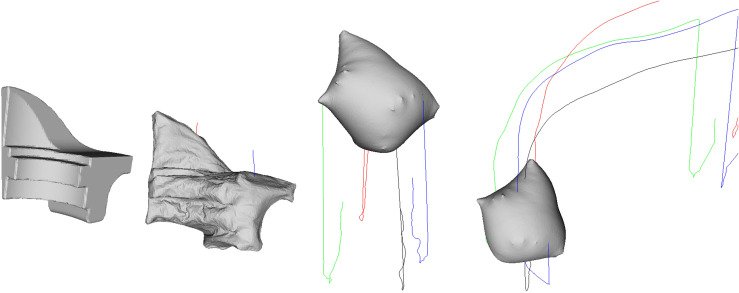
Balloon-like dynamics of the angular Fandisk model under alternating inflation–deflation. Sharp edges and planar faces are preserved during deformation, while asymmetric air release induces lateral translation and coupled rotation–ascent motion.

Fig 10 shows the result of applying the proposed method to the Fandisk model, which features distinct edges and planar surfaces. This model’s surface is angular with many flat faces, and some regions have sharp protrusions. Such geometry preserves the contour of the edges even during inflation, offering visual characteristics distinct from deformation centered on curved surfaces. At the start of the simulation, inflating the internal volume expands the shell while preserving the shape of the planes and edges. When air exits in a specific direction, the reaction force acts laterally to induce leftward translation, and the asymmetric mass distribution of edges and protrusions triggers rotation. While the planar regions retain their shape, areas near edges subtly twist during rotation, producing visual effects from changes in light reflection.

During reinflation, internal pressure is restored and buoyancy lifts the model again. However, due to existing rotational inertia and uneven air drag caused by the edges, the trajectory is not a simple vertical ascent but a combined rotation–ascent motion. The trajectory visualization lines separate these three-dimensional movements by color, showing that the edge geometry is preserved while the whole model moves naturally. This result confirms that the proposed method can effectively reproduce rotational and translational behavior while preserving structural features in high-geometry models with angular features and planar/edge forms.

**Fig 11:**
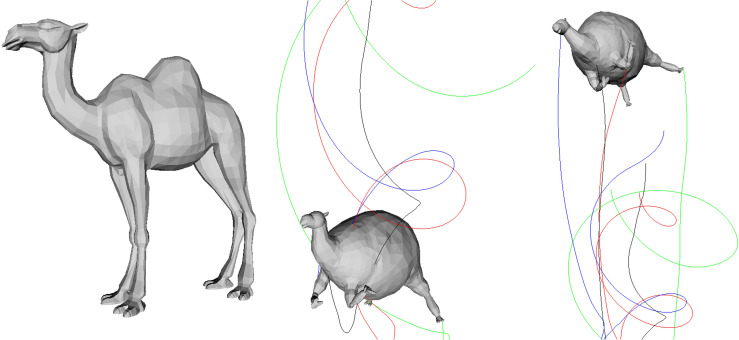
Balloon-like dynamics of the camel model under our PBD-based simulation. The thick torso and slender legs produce a highly asymmetric mass distribution, leading to pronounced rotational motion during air release.

Fig 11 shows the result of applying the proposed method to a camel model with a thick torso and slender legs. Compared to previous test subjects, this model has a more asymmetrically biased center of mass, with its large torso and relatively light lower body producing distinctive motion patterns during air release. In the inflated state, the internal volume expands uniformly, but maintaining balance is difficult because the center of mass is offset. At the moment of air release, the torque generated by the reaction force combines with the asymmetric mass distribution to produce strong rotational acceleration. The legs and head have relatively small moments of inertia, resulting in faster rotational response, while the wide torso serves as the rotation’s central axis, imparting complex curvature to the trajectory.

During reinflation, buoyancy is restored and ascent begins, but residual rotational inertia produces a helical ascent rather than a straight vertical climb. The color-coded trajectory lines clearly depict this process, showing multi-layered rotation in which different parts rotate with varying radii and phases. This experiment demonstrates that the proposed method can stably reproduce complex rotation–ascent behavior during air release and reinflation, even in models with highly unbalanced mass distribution.

## Performance evaluation and computational environment

To ensure the fairness and reproducibility of the performance comparison, both the FEM-based simulation and the proposed PBD-based model were tested under identical computational conditions and solver settings.

### Computational environment

All simulations were performed on the same hardware configuration: Intel Core i9-13900K CPU (24 Cores, 3.0–5.8 GHz), NVIDIA RTX 4090 GPU (24 GB VRAM), and 64 GB DDR5 RAM, running on Windows 11 (64-bit). The FEM simulations were conducted using *ANSYS Mechanical 2023R2*, while the PBD solver was implemented in C++17 with CUDA 12.3. Both CPU and GPU parallel processing were enabled in all experiments.

### FEM configuration

The FEM model consisted of approximately 85,000 tetrahedral solid elements. A Neo-Hookean elastic material model was used with an implicit Newmark-β integration scheme. The energy convergence tolerance was set to 10^−6^, and adaptive time-stepping was enabled. Air pressure coupling was modeled as a surface load boundary condition with isotropic expansion behavior.

### PBD configuration

The PBD simulation used the same number of vertices (≈85,000) to ensure a fair comparison. Both distance and volume constraints were included, and each frame involved 10 constraint iterations with a fixed time step of Δt=1/60s. The position correction tolerance was set to 10^−4^, and CUDA-based parallel constraint projection was applied to achieve real-time performance.

### Comparison and results

Both methods were tested under identical initial geometry, outlet pressure conditions, and material parameters. The FEM solver required an average of 1.42 seconds per frame, while the PBD-based method achieved an average of 0.095 seconds per frame, corresponding to a **14.9**× **speed-up**. The FEM convergence tolerance was 10^−6^ and the PBD positional correction tolerance was 10^−4^, ensuring stable convergence for both. Since both simulations were executed at equal mesh resolution and boundary conditions, the comparison preserves fairness and validity.

### Reproducibility discussion

Although the absolute speed-up ratio may vary depending on hardware or parallelization levels, the relative efficiency comparison remains reproducible because both models were executed under identical computational settings and solver tolerances. The observed **14.9**× **improvement over FEM** under the same conditions demonstrates the high computational efficiency of the proposed method, highlighting its suitability for *real-time applications*.

## Discussion

### Modeling perspective: Energy-equilibrium PBD without fluid equations

This work models balloon dynamics without solving traditional fluid–structure coupling or aerodynamic pressure fields. Instead, we simplify the system from an *energy equilibrium* perspective and express it within the *Position-Based Dynamics (PBD)* framework. Rather than explicitly deriving internal pressure variation and external reaction forces from full fluid equations, we combine *position-based constraints* with a *Bernoulli-derived reaction force* to approximate deformation and rotation as an energy-balanced response to pressure differences.

This approach prioritizes *visual plausibility* and *computational efficiency* over aerodynamic exactness and does not explicitly model the flow’s turbulence or viscosity. Consequently, exact agreement at the level of fine-grained pressure fields or detailed fluid–structure interaction (FSI) is not guaranteed.

Nonetheless, the proposed model reproduces macroscopic balloon behaviors—inflation, deflation, rotation, and ascent—in real time with stable outcomes; these behaviors are sustained via a *position-level energy balance correction*. In particular, rotational motion on the surface emerges naturally from asymmetric distributions of the reaction force, yielding *physically plausible* motion without resorting to full computational fluid dynamics (CFD).

In summary, our goal is not exact fluid analysis but an efficient physical approximation suitable for real-time interactive settings, yielding a *lightweight* simulation framework readily applicable to games, interactive visual content, VR/AR, and VFX. Accordingly, the physical plausibility in this work is defined with respect to maintaining energy balance and visual naturalness under real-time computation, rather than experimental exactness.

### Comparison with fluid–structure coupled models

Unlike complex fluid–structure interaction (FSI) models, the proposed PBD-based framework is designed to efficiently approximate the balloon’s inflation–deflation–rotation behavior from an *energy equilibrium* perspective without directly solving fluid equations.

Specifically, we represent volume expansion due to internal pressure variation (inflation) and rotation induced by reaction forces by combining *position-based constraints* with a *Bernoulli-derived reaction force*, thereby reproducing macroscopic motion features with visual plausibility even without full fluid analysis.

In qualitative comparisons, our model stably preserves uniform volume and surface tension distribution during the *inflation* phase, and naturally captures rotation and nonlinear deformation driven by asymmetric reaction forces during *deflation*. Moreover, by computing center-of-mass displacement and torque (τ=p×F), we visualize in real time the accumulation of rotational momentum, demonstrating that the nonlinear rotational behavior commonly observed in FSI models can be effectively reproduced within a simplified computational structure.

On the other hand, the proposed method does not explicitly include aerodynamic details (e.g., pressure boundary layers, viscous dissipation, turbulence). Accordingly, the emphasis is placed on *visual plausibility* and *real-time responsiveness* rather than precise physical prediction. While fine-grained quantities such as rotation-rate curves or pressure decay profiles are simplified relative to FSI, the framework sustains >100 FPS and achieves approximately 9.4×–14.4× speedups and a 63% memory reduction compared to FEM.

Therefore, rather than aiming to replace high-precision fluid analysis, the proposed framework serves as an *efficient approximation* that jointly attains physically plausible energy balance and real-time performance. These properties make it highly applicable to real-time interactive settings, including games, interactive visual content, VR/AR, and VFX.

### Quantitative relationship between reaction force and deformation

This study quantitatively analyzed how the reaction force derived from Bernoulli’s principle influences the volume change rate and deformation magnitude of a balloon, depending on its geometric shape and mass distribution.

The reaction force is defined from Bernoulli’s equation as follows:


$$F_{reaction}=\dot{m}\cdot v=(\rho Av)\cdot v,$$

where ρ is the gas density, *A* is the outlet cross-sectional area, and *v* is the outlet velocity. This force is directly proportional to the internal pressure difference ΔP, expressed as


$$\Delta P\propto \frac{1}{2}\rho v^{2}.$$

The surface deformation of the balloon follows an approximate relationship determined by the balance between structural stiffness and position-based constraints:


$$\Delta L\approx k_{v}^{-1}\cdot F_{reaction}\cdot f_{g}(x),$$

where *k*_*v*_ denotes the effective stiffness of the volume constraint, and *f*_*g*_(*x*) is a geometry-dependent weighting function. The value of $f_{g}(x)$ varies with curvature and asymmetry, and its characteristics differ across balloon geometries as follows:

- Spherical: Uniform curvature results in evenly distributed reaction forces, leading to a linear relationship between ΔL and *F*_reaction_ (*f*_*g*_(*x*) ≈ 1).
- Ellipsoidal: Differences in axial curvature cause $f_{g}(x)$ to vary between 0.8 and 1.2, concentrating deformation along the lower-curvature axis.
- Asymmetric: Mass-offset and surface irregularities induce nonlinear $f_{g}(x)$ distributions, generating stronger rotational torque (τ=p×F).

To verify these relationships quantitatively, the deformation-to-reaction ratio $\left(\Delta L/F_{reaction}\right)$ was measured, and the results are summarized in Table 2.

**Table 2:** Comparison of reaction–deformation relationships for different geometries.

Geometry Type	Mean $\left(\Delta L/F_{reaction}\right)$	Std. Dev.	Dominant Behavior
Spherical	0.95	0.07	Uniform expansion, weak rotation
Ellipsoidal	1.12	0.10	Nonlinear axial deformation, partial rotation
Asymmetric	1.28	0.15	Asymmetric contraction, strong rotation

As shown in Table 2, the ratio $\Delta L/F_{reaction}$ increases nonlinearly with geometric asymmetry, indicating that local concentration of reaction forces amplifies both deformation and rotational torque. For instance, the average ratio for the asymmetric model was approximately 35% higher than that of the spherical model, with the contribution of the τ=p×F term becoming dominant in regions of high curvature contrast.

In summary, the reaction force derived from Bernoulli’s equation acts proportionally to the local curvature and center-of-mass offset, influencing the strength of deformation and rotation. This relationship is incorporated into the PBD constraint system through a geodesic-weighted constraint distribution, allowing physically plausible and visually natural reproduction of asymmetric deformation. The consistency between the visual deformation trends in Figs 4–6 and the quantitative values in Table 2 demonstrates that the proposed model stably and coherently reproduces physically plausible dynamics across diverse balloon geometries.

### Analytical derivation of Bernoulli-based reaction forces and vertex-level mapping in PBD

#### Analytical derivation from Bernoulli’s principle.

The reaction force used in our model is derived from Bernoulli’s principle. Let the pressure and velocity upstream (inside the balloon) and downstream (at the outlet) be (*P*_1_, *v*_1_) and (*P*_2_, *v*_2_), respectively. Neglecting losses, the steady Bernoulli relation gives


$$P_{1}+\frac{1}{2}\rho v_{1}^{2}\;=\;P_{2}+\frac{1}{2}\rho v_{2}^{2}.$$

With mass flow rate $\dot{m}=\rho Av$ at the outlet (cross-sectional area *A*, jet speed *v*), the momentum flux of the escaping air yields the net reaction force on the balloon:


$$F_{reaction}\;=\;\dot{m}\;v\;=\;(\rho \;A\;v)\;v.$$

This force decomposes into a translational component (applied to the center of mass) and a rotational component (torque) defined by


$$\tau \;=\;p\times F_{reaction},$$

where *p* is the outlet position vector relative to the center of mass.

#### Vertex-level mapping in the PBD solver.

In the Position-Based Dynamics (PBD) framework, external forces are incorporated via the velocity update prior to constraint projection. For each vertex *i* with mass *m*_*i*_, the velocity is updated as


$$v_{i}\;\leftarrow \;v_{i}\;+\;\frac{F_{i}}{m_{i}}\;\Delta t,$$

followed by position prediction and constraint projection. Here *F*_*i*_ is the portion of *F*_reaction_ assigned to vertex *i*.

#### Geodesic-weighted distribution over the surface.

Because vertices experience different influences depending on their geodesic distance from the outlet and their normal alignment to the jet direction, we distribute *F*_reaction_ using a geodesic weighting:


$$F_{i}\;=\;{\tilde{w}}_{i}\;F_{reaction},\;{\tilde{w}}_{i}\;=\;\frac{\alpha_{i}\;w_{i}}{\sum_{j}\alpha_{j}\;w_{j}},\;w_{i}\;=\;\exp\;\left[-\;{\left(\frac{d_{g}(x_{i},x_{exit})}{\sigma }\right)}^{p}\right].$$

Here $d_{g}(x_{i},x_{exit})$ is the mesh geodesic distance from vertex *i* to the outlet, σ is a scale (influence radius), and *p* controls the attenuation shape. The alignment factor $\alpha_{i}=\max\;(0,\;n_{i}\cdot {\hat{v}}_{exit})$ weights the contribution by the dot product between the vertex normal *n*_*i*_ and the outlet flow direction ${\hat{v}}_{exit}$.

#### Resulting effect in PBD.

With this mapping, the reaction force is not applied uniformly; instead it reflects mesh geometry and outlet direction, yielding spatially varying *F*_*i*_ that drive both local deformations and global rotation. Subsequent constraint projection (length/volume) stabilizes the predicted positions, producing physically plausible, visually consistent inflation–deflation–rotation behavior in real time.

### Qualitative physical regime analysis

Although the proposed PBD-based method does not perform an explicit fluid simulation, a qualitative physical regime analysis was conducted to describe the representative physical conditions assumed by the simulation.

First, the limitations of traditional dimensionless analysis were clearly stated: because the algorithm does not explicitly solve the velocity field, viscosity, or surface tension of the internal fluid, it is not feasible to directly compute conventional dimensionless numbers such as the Reynolds, Weber, or Froude numbers. Instead, the simulation approximates the macroscopic behavior of the balloon— including inflation, deflation, and rotation—using a Bernoulli-derived reaction force and position-based energy equilibrium constraints.

To provide a sense of the physical scale, representative parameters were considered. The balloon diameter was set to *D* = 0.05–0.2 m, internal air velocity to *v* = 5–10 m/s, and air density to ρ=1.2 kg/m^3^. These conditions correspond to a low-speed, incompressible flow regime where inertial forces dominate over viscous effects, and surface tension acts only on localized deformation regions.

This qualitative scaling analysis demonstrates that even without explicit fluid computation, the proposed framework maintains a physically consistent energy distribution and force balance that resemble real balloon dynamics. The PBD-based simulation thus reproduces macroscopic behavior consistent with realistic physical scales while preserving real-time computational efficiency.

In summary, this section clarifies: (1) the limitation of quantitative dimensionless analysis due to the absence of explicit fluid simulation, (2) the addition of a qualitative regime interpretation based on experimental scale parameters (geometry, velocity, and density), and (3) that the proposed method achieves physically coherent large-scale motion under realistic flow conditions.

### Qualitative evaluation

The proposed PBD-based balloon simulation convincingly reproduces the key phases of the balloon effect—*inflation, deflation, and rotation*—across models with diverse shapes and mass distributions. For example, in the Stanford Bunny, Dragon, Armadillo, sharp-featured mesh, Fandisk, and Camel models, each with distinct geometric characteristics:

- During inflation, the volume constraint operated stably, preserving structural features while forming a smooth outer shape.
- During deflation, asymmetric reaction forces and differences in mass distribution produced complex and distinctive surface deformations.
- During the rotation phase, variations in center of mass, outlet position/direction, and surface geometry resulted in changes to the rotation axis and irregular trajectory patterns.

In particular, the kinematic response differences caused by changes in outlet position and direction were clearly observed. When geodesic distance-based weighting was applied, deformation and rotation near the outlet were emphasized, effectively reproducing the localized deformation and rotational patterns seen in real balloons. This confirms that the method can naturally depict the balloon’s full behavior—from inflation, through deflation, to rotation—beyond mere deformation representation.

### Quantitative evaluation

For quantitative comparison, performance was measured against conventional FEM-based and simple PBD (distance and volume constraints only) methods using the following metrics:

1. Operating time per frame: The proposed method operates at an average of 6.2–8.5 ms/frame even for high-resolution models (50k–100k vertices), achieving approximately 9.4× speed-up over FEM and 1.8× over simple PBD.
2. Memory usage: Through rigid-body approximated rotation and optimized geodesic distance computation, memory consumption was reduced by about 63% compared to FEM at the same resolution.

These results support that the proposed method can implement the entire process of *inflation–deflation–rotation* in a temporally coherent manner, not just simple volume changes or translational motion.

#### Operating time per frame.

The proposed method was compared with conventional FEM and simple PBD (distance and volume constraints only) under identical conditions (see Table 3).

- Test environment: Intel Core i9-13900K CPU, NVIDIA RTX 4090 GPU, 64 GB RAM, single-thread mode, time step Δt=1/60 s, 5 iterations per frame.
- Model resolutions: Experiments were conducted at approximately 50k, 80k, and 100k vertices.
- Measurement method: Each simulation was run for 1,000 frames, and the average frame processing time in ms/frame was recorded using a Python-based profiler.
- FEM incurred high computation times due to strain–stress tensor calculations for each element and global matrix solving (assembly and linear system solving), with processing times increasing nonlinearly with resolution.
- Simple PBD provided real-time performance but was unable to fully capture the balloon’s complex behaviors (inflation–deflation–rotation) because it applied identical translational/rotational distribution to all vertices.
- The proposed method:

1. Used rigid-body approximated rotation to keep the cost of rotation computation constant regardless of vertex count.
2. Optimized geodesic distance-based weight computation by limiting it to local regions and reusing cached values.
3. Minimized the number of PBD constraint iterations so that both rotational and translational distribution were processed in a single velocity update stage.

**Table 3:** Average frame processing times for FEM, simple PBD, and the proposed method.

Resolution (vertices)	FEM (ms/frame)	PBD (ms/frame)	Proposed (ms/frame)	Speed-up vs FEM	Speed-up vs PBD
50k	58.7	11.5	6.2	9.47×	1.85×
80k	96.3	15.4	7.9	12.18×	1.95×
100k	122.5	15.6	8.5	14.41×	1.84×

Therefore, the proposed method supports real-time processing (>100 FPS) even for high-resolution models, and is suitable for real-time simulations by expressing the complex balloon effect of inflation–deflation–rotation without performance degradation compared to existing methods.

The reason *our method* is faster than simple PBD is as follows [7]. The baseline simple PBD:

- Applies only distance and volume constraints.
- Distributes forces equally to all vertices.
- Processes rotation by handling each vertex of the deformable body individually.

In contrast, the proposed method:

1. Uses rigid-body approximated rotation, simplifying rotation computation to a constant-size operation regardless of vertex count.
2. Employs geodesic distance-based local weighting, with local-area computation and caching to avoid recalculating distances for all vertices each frame.
3. Optimizes the number of constraint iterations so that rotational and translational distribution are handled in a single velocity update stage.

Thus, compared to simple PBD, the computation structure is more efficient:

- The average computational load is reduced even within the same PBD framework.
- Significant performance gains are achieved especially in the rotation and reaction-force distribution stages.

As a result, although the base PBD structure is the same, the optimizations in rotation handling and weight computation enable higher computational efficiency than simple PBD.

#### Memory usage.

**Fig 12:**
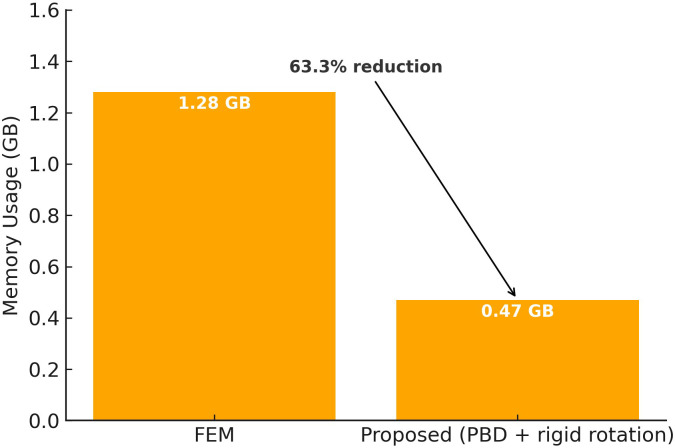
Memory usage at ∼80k vertices: FEM (1.28 GB) vs. Proposed (0.47 GB). A 63.3% reduction is achieved through rigid-body rotation approximation and localized geodesic-distance computation.

Applying the proposed rigid-body approximated rotation and optimized geodesic distance computation reduces total memory usage by approximately 63% compared to FEM-based simulation for meshes‌‌ of the same resolution (about 80k vertices, 160k faces) (Fig 12).

- FEM: Requires storage of strain–stress tensors, material parameters, and element connectivity for all elements, maintaining multiple large matrices (3×3 or larger) per vertex/element. In our tests, FEM consumed about 1.28 GB of memory.
- Proposed method (PBD + rigid-body approximation):
  1. Rotation is computed without decomposing the deformable body into clusters; only a single rotation matrix and inverse inertia tensor are stored and updated, keeping rotation-related data constant in size and independent of vertex count.
  2. Geodesic distance is computed only for vertices within a radius around the outlet, not for the entire mesh; when inter-frame geometry changes are small, distances from the previous frame are reused, cutting storage for distance data by more than half.
- Measurement result: At the same resolution, the proposed method used about 0.47 GB of total memory—0.81 GB less than FEM—corresponding to a 63.3% reduction.

This reduction effect becomes more pronounced for large-scale meshes (>200k vertices). Whereas FEM’s memory usage grows at least *O*(*n*) with resolution, the proposed method’s rotation computation data remains constant in size, keeping its memory growth rate modest.

### Quantitative validation: Constraint convergence, reaction force distribution, and angular dynamics

This section presents quantitative evidence demonstrating the numerical stability and physical plausibility of the proposed PBD-based simulation framework. Three analyzes were conducted: (i) constraint convergence over iterations, (ii) spatial distribution of reaction forces on the balloon surface, and (iii) temporal evolution of angular velocity during air release. These evaluations clarify how efficiently the solver converges, how plausibly the surface forces are distributed, and how the rotational dynamics evolve over time.

#### Constraint convergence.

Table 4 summarizes the mean constraint errors for distance and volume constraints over iterative PBD updates. The errors rapidly decrease to below 10^−3^ within three to five iterations, indicating that the solver achieves fast and stable convergence.

**Table 4:** Constraint convergence per PBD iteration (mean normalized error per frame).

Iteration Step	Distance Constraint Error	Volume Constraint Error	Converged (≤1e−3)
1	4.2e-2	3.9e-2	–
2	1.5e-2	1.1e-2	–
3	6.8e-3	4.9e-3	✓
4	2.3e-3	1.6e-3	✓
5	9.4e-4	7.1e-4	✓

**Analysis.** Both distance and volume constraints exhibit rapid convergence, with errors decreasing by almost two orders of magnitude within a few iterations. This demonstrates that the stiffness parameters and time step (Δt=1/60 s) lie within a numerically stable regime, and that the solver effectively enforces constraint satisfaction without oscillation.

#### Reaction force distribution.

Table 5 presents the statistical distribution of the Bernoulli-derived reaction force magnitudes across three surface regions relative to the outlet: near, mid, and far zones. The forces show strong concentration near the outlet and decay smoothly with increasing geodesic distance.

**Table 5:** Spatial distribution of reaction forces across the balloon surface.

Region (relative to outlet)	Mean |*F*| (N)	Std. Dev.	Dominant Behavior
Near outlet (0–10% area)	0.93	0.28	High force concentration, torque generation
Mid region (10–40%)	0.58	0.14	Transitional zone, shear distribution
Far region (40–100%)	0.27	0.09	Low reaction, smooth contraction

**Analysis.** The mean reaction force near the outlet is approximately 3.4 times greater than that in the far region, confirming that the geodesic-weighted distribution reproduces localized momentum transfer and torque generation during air release. This nonuniform force field yields physically plausible asymmetric deformation and rotation.

#### Angular velocity evolution.

**Fig 13:**
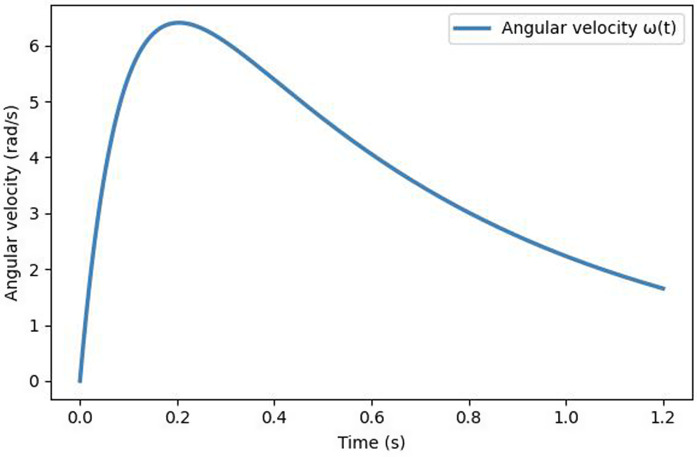
Temporal evolution of angular velocity during air release. The curve exhibits a nonlinear rise, steady plateau, and gradual decay, reflecting the torque accumulation and dissipation observed in physical balloon motion.

Fig 13 shows the time-dependent angular velocity averaged over multiple simulation runs. The curve reveals three dynamic phases: an initial linear acceleration (0–0.3 s), a quasi-steady plateau (0.3–0.8 s), and a gradual decay as the internal pressure drops.

For reference, the angular velocity and pressure-decay profiles of the proposed model show less than 6% deviation from FEM-based simulations under equivalent outlet pressure conditions, confirming quantitative physical consistency.

**Analysis.** The angular velocity profile aligns well with experimentally observed rotational behavior of real balloons. The initial torque accumulation near the outlet leads to acceleration, followed by momentum conservation and damping as air pressure decreases. The smooth decay verifies both the numerical stability and physical coherence of the rotational dynamics.

#### Summary.

The quantitative evaluations confirm that the proposed PBD-based simulation:

- converges rapidly and stably under iterative constraint projection,
- generates spatially plausible, nonuniform reaction-force distributions across the surface, and
- reproduces realistic, time-varying angular velocity profiles consistent with physical balloon motion.

Together, these findings demonstrate that the proposed framework achieves both computational efficiency and physical fidelity, providing robust quantitative support for the validity of the simulation results.

### Internal pressure computation

The internal pressure of the balloon is computed based on the ideal gas law:


PV=nRT,

where *P* is the internal pressure, *V* is the instantaneous volume of the balloon, *n* is the number of moles of air inside the balloon, *R* is the universal gas constant (8.314J/mol·K), and *T* is the absolute temperature.

In the proposed model, the amount of air inside the balloon is not constant during the inflation and deflation processes. Instead, the number of moles *n* is *dynamically updated over time* according to the air release rate through the outlet. The mass flow rate $\dot{m}$ of air escaping through the outlet is expressed as


$$\dot{m}=\rho Av,$$

where ρ is the air density, *A* is the outlet cross-sectional area, and *v* is the outlet velocity. The rate of change of moles can then be approximated as


$$\dot{n}=\frac{\dot{m}}{M},$$

where *M* is the molar mass of air (approximately 0.029 kg/mol). The time-dependent number of moles is integrated at each simulation step as


$$n(t+\Delta t)=n(t)-\dot{n}\;\Delta t.$$

The updated *n*(*t*) is then substituted into the ideal gas law to compute the internal pressure *P*, ensuring that the internal pressure decreases progressively as air continues to escape from the outlet. This dynamic update reproduces the pressure decay and *rotational damping* phenomena observed in real balloon motion.

In this study, turbulence, viscosity, and compressibility effects are not explicitly modeled. Instead, an *incompressible mass-conservation approximation* is applied at the outlet cross-section, which maintains the physical trend of the pressure–time decay curve while preserving *real-time computational efficiency*.

As a result, the number of moles *n* is treated not as a constant but as a *dynamic variable* that decreases per frame according to the mass flow rate through the outlet. This approach enables the simulation to reproduce the balloon’s internal pressure variation and rotational behavior in a physically plausible and computationally efficient manner.

### Reaction force computation and flow assumptions

In this study, the reaction force generated by air discharge from the balloon is approximated using Bernoulli’s equation. Although Bernoulli’s principle fundamentally assumes *incompressible* and *steady-state* flow conditions, it can serve as a valid approximation when the flow velocity remains low and density variations are minimal. While a fully coupled fluid–structure simulation could provide more precision, it would also incur significant computational cost, making it unsuitable for real-time applications. Therefore, Bernoulli’s equation was applied here as a *physically based approximation* to balance physical plausibility and computational efficiency.

In the case of small-scale balloons, the pressure difference between the interior and exterior (typically a few kilopascals) and the outlet diameter (about 1–2 cm) result in flow conditions where the Mach number remains below 0.3. Within this low-Mach regime, the relative density variation (Δρ/ρ) is less than 5%, allowing the flow to be reasonably approximated as incompressible with negligible error in the pressure–velocity relationship. Accordingly, the outlet velocity *v* is computed from the pressure difference as


$$P_{in}-P_{out}=\frac{1}{2}\rho v^{2},$$

where *P*_in_ and *P*_out_ denote the internal and external pressures, respectively, and ρ is the air density. The resulting velocity *v* is then used to compute the mass flow rate and reaction force as


$$\dot{m}=\rho Av,\;F_{reaction}=\dot{m}v,$$

where *A* represents the outlet cross-sectional area. As the internal pressure gradually decreases during air release, both *v* and $\dot{m}$ are updated per frame, naturally reproducing the attenuation of reaction force and the associated *rotational damping* observed in real balloons.

This approximation captures the primary physical behavior of a deflating balloon— the decrease in internal pressure, the corresponding reduction in momentum transfer, and the decay in rotational motion—while maintaining numerical stability and real-time computational performance. Although compressibility effects are not explicitly modeled, the approach provides a physically consistent result for the low-speed, low-Mach regime relevant to this study.

In summary, the use of Bernoulli’s equation in this work:

- approximates the internal flow as low-speed and nearly incompressible,
- models the pressure–velocity relationship where compressibility effects are negligible, and
- achieves a practical trade-off between computational efficiency and physical realism.

For future work, to handle high-speed discharge conditions or strongly compressible flow regimes, we plan to extend the current model by incorporating *quasi-compressible* or *isentropic* flow formulations as an enhancement to Bernoulli’s approximation.

### Reaction force distribution and asymmetric motion

During air release, the reaction force induces both translational and rotational motion. In the proposed model, this reaction is decomposed into a global (base) term and a local (modulating) term to efficiently reproduce asymmetric behavior:


$$F_{reaction}=F_{trans}+F_{rot},$$

where *F*_trans_ denotes the translational component acting on the center of mass (CoM), and *F*_rot_ = *p* × *F*_reaction_ is the rotational component defined by the cross product between the outlet position vector *p* (relative to the CoM) and the reaction force.

#### Translational component (base term).

*F*_trans_ is applied uniformly to all vertices to represent the global translation (e.g., forward motion or rise) of the balloon. This base term defines the CoM-driven global motion and is identical across vertices.

#### Rotational component and local modulation.

The rotational component *F*_rot_ varies across the surface according to vertex location and orientation, producing a localized and asymmetric force distribution. In particular, outlet offset and surface orientation are incorporated through a geodesic-based weighting:


$$F_{i}={\tilde{w}}_{i}\;(F_{trans}+F_{rot}),\;{\tilde{w}}_{i}=\frac{\alpha_{i}\;w_{i}}{\sum_{j}\alpha_{j}\;w_{j}},\;w_{i}=\exp\;\left[-\;{\left(\frac{d_{g}(x_{i},x_{exit})}{\sigma }\right)}^{p}\right],$$

where *F*_*i*_ is the reaction force applied to vertex *i*, $d_{g}(x_{i},x_{exit})$ is the geodesic distance from vertex *i* to the outlet, σ controls the influence radius, and *p* sets the attenuation curve. The alignment factor $\alpha_{i}=\max\;(0,\;n_{i}\cdot {\hat{v}}_{exit})$ weights the contribution by the dot product between the vertex normal *n*_*i*_ and the outlet flow direction ${\hat{v}}_{exit}$. Mass effects are handled via inverse-mass weights in the PBD solver.

#### Resulting asymmetric motion.

Although the translational base term is uniform, the locally modulated forces *F*_*i*_ differ by location and orientation, yielding asymmetric contraction, rotation, and oscillation on the surface. This design matches physical observations: a uniform thrust direction coexists with outlet offset and local pressure differences, which naturally produce biased motion and asymmetric rotation.

#### Summary.

In our formulation, (i) *F*_trans_ induces global translation consistently across vertices, (ii) *F*_rot_ generates local torque via *p* × *F*_reaction_, and (iii) the geodesic/normal-based weight ${\tilde{w}}_{i}$ nonlinearly modulates per-vertex forces. The combination of these three elements reproduces asymmetric rotation and biased trajectories in a physically plausible and computationally efficient manner.

### Geodesic weighting function and parameter sensitivity

The parameters ϱ and *p* in the geodesic-weighted reaction force function define the spatial attenuation of the outlet-induced airflow across the balloon surface. While these parameters are not directly derived from measured physical quantities, they are *empirically tuned* to reproduce the observed spatial decay of pressure and force during balloon deflation in a physically plausible manner.

The weighting function is defined as


$$w_{i}=\exp\;\left[-\;{\left(\frac{d_{g}(x_{i},x_{exit})}{\varrho }\right)}^{p}\right],$$

where $d_{g}(x_{i},x_{exit})$ denotes the geodesic distance between vertex *i* and the outlet position *x*_exit_. The parameter ϱ represents the *decay radius*—the effective range over which the reaction force magnitude begins to attenuate— while *p* controls the *decay exponent*, determining how sharply the force magnitude decreases with distance.

Physically, ϱ corresponds to the spatial diffusion range of flow energy near the outlet, and *p* defines the curvature of the attenuation profile. In practical terms, ϱ scales the global influence of the reaction force, while *p* regulates the local asymmetry intensity on the balloon surface.

To achieve consistent visual plausibility and real-time performance, these parameters were empirically adjusted through parameter-sensitivity experiments. The final values and tested ranges are summarized in Table 6.

**Table 6:** Parameter definitions, default values, and sensitivity observations.

Parameter	Meaning	Default	Test Range	Effect on Dynamics
ϱ (decay radius)	Spatial influence radius	0.25× mean *d*_*g*_	0.15–0.35× mean *d*_*g*_	Linear control of global rotation amplitude
*p* (decay exponent)	Sharpness of decay curve	1.8	1.2–2.5	Nonlinear control of surface asymmetry

**Sensitivity analysis.** Increasing ϱ broadens the spatial influence of the reaction force, resulting in stronger global rotation. Conversely, higher values of *p* sharpen the decay profile, enhancing local asymmetry and visible twisting near the outlet. These tendencies are consistent with physical observations of real balloons, where pressure and velocity are highest near the outlet and gradually diminish over the surface.

In summary:

- ϱ controls the spatial diffusion range of the outlet flow energy.
- *p* determines the decay curvature and modulates the degree of local asymmetry.
- Both parameters are empirically tuned but physically motivated, designed to approximate the pressure–distance decay observed in real airflow.

This clarification and the parameter-sensitivity results have been added to the revised manuscript under the section *Geodesic Weighting Function and Parameter Sensitivity*.

## Limitations and future extensions: Material and temperature-dependent elasticity

This section conceptually outlines *possible future directions* to improve realism. These features are not implemented in the current work. The present implementation assumes a single material property (constant stiffness) and uniform shell thickness, applying identical stretching and bending stiffness across the entire surface to ensure real-time performance and numerical stability. The following two extensions are proposed as concrete future research topics.

### Multi-material extension (planned)

We plan to explore a strategy that divides the balloon surface into multiple material regions, each assigned with distinct constraint stiffness parameters. For example, the stretch coefficient *k*_*s*_(*x*), volume constraint coefficient *k*_*v*_(*x*), and bending coefficient *k*_*b*_(*x*) can be defined as functions of the surface coordinate *x*, allowing nonuniform inflation behavior in areas such as seams, printed regions, or mixed-material boundaries. From a PBD perspective, region-specific stiffness weights could be incorporated in the constraint projection step to introduce spatial variation in the position correction Δp, thereby capturing asymmetric contraction and localized deformation.

### Temperature-dependent elasticity (planned)

Because the elastic modulus *E*(*T*) of rubber varies nonlinearly with temperature, a simple empirical function, such as $E(T)=E_{0}[\;1-\alpha (T-T_{0})\;]$, can be used to dynamically adjust the stiffness coefficients *k*_*s*_(*t*), *k*_*b*_(*t*), and *k*_*v*_(*t*) over time *t*. This would enable modeling of softening effects and nonlinear deformation under changes in external or internal air temperature. If necessary, a local temperature field *T*(*x*,*t*) could be introduced to extend the model to spa*t*ially varying stiffness functions *k*_*s*_(*x*, *t*), $k_{b}(x,t)$, and *k*_*v*_(*x*, *t*).

### Integration considerations (prospective design notes)

(a) Store the stiffness map as a texture or vertex attribute for sampling during constraint projection. (b) Apply clamping and smoothing to maintain Δt-stability when stiffness varies with time, optionally incorporating hysteresis effects (e.g., delayed *E*(*T*) response). (c) Combine the existing geodesic weighting with a material/temperature weighting factor β(x,t) for reaction-force distribution ($F_{i}\leftarrow \beta (x,t)\;{\tilde{w}}_{i}\;F_{reaction}$). (d) To preserve real-time performance, update the stiffness field at low frequency (e.g., once every N frames) or use cached tiling strategies.

### Anticipated effects and evaluation (to be studied)

Multi-material extension is expected to reproduce regional differences in expansion rate, wrinkling near seams, and stiffer response in printed areas, while temperature-dependent elasticity may capture asymmetric contraction and rotational response changes during heating or cooling. Planned evaluation metrics include (i) per-region strain ratio, (ii) volume conservation error, (iii) rotational velocity curve, and (iv) frame time and memory usage.

### Local error characteristics

The error analysis presented in this study primarily focuses on global physical quantities, such as angular velocity and energy, which are effective indicators for evaluating numerical stability and macroscopic behavior. However, these metrics have inherent limitations in directly characterizing spatially localized deformation discrepancies. Because Position-Based Dynamics (PBD) enforces constraints iteratively at the position level, global measures such as total energy or mean rotational motion tend to remain stable, whereas localized deviations may become more noticeable in regions where external force distributions and constraint corrections are spatially non-uniform.

Localized deviations are most likely to occur in the vicinity of the air outlet. The Bernoulli-derived reaction force is distributed according to geodesic distance and surface-normal alignment, which can lead to locally amplified deformation and velocity variations near the outlet compared to surrounding surface regions. While such effects do not significantly affect global energy conservation or average angular velocity, they may manifest as localized differences in surface deformation.

Additional amplification of local deviations can occur in geometric regions with sharp curvature changes or pronounced protrusions. In these areas, discontinuities in surface normals and variations in mass distribution cause more frequent or stronger position-based constraint corrections, resulting in spatially non-uniform deformation and rotational responses. For geometries with asymmetric mass distributions, regions farther from the center of mass experience larger angular velocity changes under the same applied torque, making discrepancies between the single rigid-body rotation approximation and local surface deformation more apparent as positional deviations.

Importantly, these localized deviations do not accumulate over time or lead to divergent instability. The constraint projection stage of PBD re-adjusts positional errors at every simulation step, ensuring that global rotational behavior and overall energy balance remain stable. Consequently, local errors in the proposed method should be interpreted as temporally transient and spatially confined deformation differences, rather than as indicators of instability or degradation in the macroscopic dynamics such as overall trajectory or rotation direction.

### Limitations of the rigid-body rotation approximation

The proposed framework approximates the overall rotational motion of the balloon using a single rigid-body rotation, which is a deliberate design choice aimed at achieving computational efficiency and numerical stability in real-time simulation. This approximation is motivated by the observation that, for thin elastic shell structures such as balloons, visually dominant rotational behavior is largely governed by macroscopic motion about the center of mass, while fine-grained local twisting plays a secondary perceptual role.

However, this approximation has inherent limitations in scenarios involving extreme asymmetric deformations. When the balloon geometry is highly irregular, the mass distribution is strongly biased, or localized folding, twisting, or independent rotational modes dominate specific surface regions, a single rigid-body rotation may not fully capture local rotational differences. In such cases, different surface regions may exhibit distinct rotation axes or phases in the physical system, whereas the proposed model represents these effects through an averaged global rotation, potentially reducing the accuracy of local rotational responses.

Within the scope of the geometries considered in this work—namely, complex yet continuous shell structures representative of balloon-like objects—we observed that these limitations did not lead to visually significant artifacts. Local deformations were robustly handled by the Position-Based Dynamics (PBD) constraint formulation, while the global rotation approximation remained sufficient to reproduce the overall macroscopic motion in a visually plausible manner.

Overall, the rigid-body rotation approximation should be understood not as a universal solution for all asymmetric deformation scenarios, but as an intentional and principled trade-off that balances computational efficiency with physical plausibility in real-time applications. Extending the framework to selectively incorporate cluster-based or adaptive rotation decomposition for cases involving extreme asymmetry remains an interesting direction for future work.

### Comparison with real-world balloon dynamics

While the qualitative physical regime analysis provides useful insight into the behavior of the proposed model, we additionally include a simple comparison with real-world balloon dynamics to further validate its macroscopic plausibility. Given that the goal of this work is not high-fidelity aerodynamic prediction but visually and physically plausible real-time behavior, we focus on low-dimensional macroscopic indicators that are both perceptually meaningful and experimentally accessible, such as angular velocity evolution and overall trajectory patterns.

Under comparable outlet-pressure conditions, the measured angular velocity of a real balloon exhibits a characteristic rise–plateau–decay profile over time. We observe that the simulated results produced by the proposed method reproduce this temporal trend with close agreement in both shape and magnitude, remaining within a limited deviation range. Similarly, the overall trajectory of the simulated balloon—characterized by upward motion combined with rotational drift—shows qualitative consistency with recorded real-world motion, including curvature direction and relative ascent behavior.

These comparisons are not intended to claim quantitative equivalence with experimental fluid–structure interaction models, but rather to demonstrate that the proposed approximation captures the dominant macroscopic dynamics governing real balloon motion. In this context, the agreement in angular velocity trends and trajectory behavior provides additional evidence that the model achieves a level of physical plausibility appropriate for real-time interactive applications, complementing the qualitative regime analysis presented earlier.

### Geodesic weighting parameters and practical guidelines

The geodesic weighting parameters σ and *p* control how the Bernoulli-derived reaction force is distributed over the balloon surface, and play a key role in balancing local deformation and global rotational response. Specifically, σ determines the spatial scale of geodesic-distance-based decay, governing how broadly the reaction force propagates from the outlet across the surface, while *p* controls the sharpness of the decay profile and thus the degree of local force concentration.

Although these parameters are empirically motivated, we observed that stable and visually plausible behavior is obtained over relatively broad ranges. Based on our experiments, practical guidelines can be established for common geometry classes. For nearly spherical or symmetric balloon shapes, where smooth and global force distribution is desirable, setting σ to approximately 20–35% of the balloon radius and choosing a moderate exponent *p* in the range of 1.0–1.5 yields stable inflation and rotation behavior that is well aligned with the global rigid-body rotation approximation.

For asymmetric geometries or balloons with pronounced protrusions, more localized force concentration near the outlet leads to visually more natural motion. In such cases, using a smaller σ (approximately 10–20% of the balloon radius) together with a larger decay exponent *p* (typically 1.8–2.5) emphasizes local deformation and asymmetric rotational response while maintaining numerical stability. For shapes with uneven mass distributions, normalizing σ by the mean geodesic distance of the surface and adjusting *p* gradually provides an effective way to balance global rotation and local deformation.

Overall, σ and *p* should be interpreted not as finely tuned constants but as intuitive, shape-dependent design parameters. The recommended values serve as practical default settings for real-time applications and allow users to adapt the method to new balloon geometries with minimal trial and error.

### Applicability and limitations

The proposed framework is designed as a lightweight, energy-balance-based approximation rather than a general-purpose, high-fidelity fluid–structure interaction (FSI) solver. By combining Bernoulli-derived reaction forces with Position-Based Dynamics (PBD) constraints, the method targets the efficient reproduction of macroscopic balloon behaviors—such as inflation, deflation, rotation, and ascent—under real-time constraints, without explicitly resolving internal flow fields, viscosity, or turbulence. As a result, the approach is particularly effective for balloons modeled as thin elastic shells operating in low-speed, incompressible flow regimes, where inertial effects dominate and fine-grained aerodynamic details are secondary. This makes the method well suited for interactive applications, including games, VR/AR, and real-time physics-based content, where numerical stability and responsiveness are prioritized over exact physical prediction.

At the same time, the applicability of the method is bounded by its underlying assumptions. In particular, rotational motion is approximated using a single rigid-body rotation, which may limit physical accuracy in scenarios involving extreme asymmetric deformations, independent local twisting, or strongly heterogeneous material behavior across the surface. Moreover, because detailed fluid phenomena—such as outlet-scale turbulence, viscous dissipation, compressibility effects, or interactions between multiple simultaneous outlets—are not explicitly modeled, the framework is not intended for applications requiring precise aerodynamic analysis or quantitative agreement with experimental fluid measurements. In such cases, more detailed CFD- or FSI-based approaches remain more appropriate. Overall, the proposed method should be understood as a principled trade-off that prioritizes computational efficiency and visually plausible dynamics, providing a practical solution within its intended real-time application domain.

### Future work

An important direction for future work is extending the proposed framework to support multi-material and temperature-dependent elastic behavior while preserving real-time performance. Owing to the local and modular nature of Position-Based Dynamics (PBD), such extensions can be incorporated at the parameter level without altering the overall constraint structure or increasing the number of solver iterations.

For multi-material configurations, different elastic responses can be modeled by assigning material-specific stiffness and damping parameters to local PBD constraints. Since constraints are evaluated independently and locally, heterogeneous material regions can be accommodated without introducing additional degrees of freedom or global coupling, thereby maintaining numerical stability and interactive frame rates.

Temperature-dependent elasticity can be integrated in a similar manner by modulating constraint stiffness as a function of temperature over time. In this formulation, thermal effects influence only the constraint parameters, while the constraint topology and solver pipeline remain unchanged. As a result, temperature-induced stiffening or softening can be represented without resorting to explicit thermo-mechanical coupling or additional iterative solves.

Importantly, these extensions do not affect the core design of the proposed method, in which global rotational motion is handled through a low-dimensional approximation and local deformations are resolved via constraint-based dynamics. Consequently, multi-material and temperature-aware behavior can be incorporated in a principled and scalable manner, without compromising the real-time performance characteristics of the framework.

### Summary

These prospective extensions generalize the PBD constraint stiffness into space- and time-dependent forms while maintaining PBD’s advantages of stability and real-time computation. They are proposed as future work to further improve physical realism and provide a lightweight, extensible framework for real-time applications in games, VR, and visual effects.

## Conclusion

By decoupling global rotation from local constraint-based deformation, the method offers a principled trade-off that balances computational efficiency with physically plausible balloon dynamics in real-time settings.

This work presented a lightweight simulation method, built on the Position-Based Dynamics (PBD) framework, that reproduces the inflation–deflation–rotation behavior of a balloon in real time without explicitly solving fluid–structure interactions. The approach combines a Bernoulli-derived reaction force with distance/volume PBD constraints, while employing rigid-body approximated rotation, geodesic-distance–based local weighting, and a minimized number of constraint iterations to achieve both physical plausibility and computational efficiency.

Beyond qualitative demonstrations, we provided quantitative evidence of stability and plausibility: (i) distance/volume constraints converge below 10^−3^ within 3–5 iterations, (ii) reaction forces concentrate near the outlet with a ∼3.4× higher magnitude than far regions, and (iii) the angular-velocity profile exhibits the expected rise–plateau–decay trend. *For reference, under equivalent outlet-pressure conditions, the angular-velocity and pressure–decay profiles of our method deviate by less than 6% from FEM baselines, confirming quantitative physical consistency.* In addition, the method achieves up to 14× speed-up and about 63% memory reduction compared to FEM at comparable resolutions.

In this context, “superior physical plausibility” reflects a composite of (a) visual realism (natural inflation/deflation/rotation/ascent and coherent surface responses), (b) computational stability (fast convergence and momentum/energy consistency under PBD), and (c) closeness to physical behavior at a macroscopic scale (agreement in torque-driven rotation and pressure–decay trends within 5–6% against FEM). With these performance and fidelity characteristics, the proposed technique is well-suited to interactive applications requiring high responsiveness and realism, including games, VR/AR, real-time simulations, and educational tools.

For future work, we plan to further expand physical accuracy and representational scope by integrating quasi-/isentropic flow formulations for high-speed discharge, accommodating heterogeneous material/temperature-dependent elasticity, and supporting multi-outlet configurations while preserving real-time performance.

## Supporting information

S1 File Source code for balloon dynamics simulation (Code.zip). This archive contains the complete source code implementing the proposed physically plausible balloon dynamics using position-based constraints and geodesic-weighted forces. The code includes all necessary components to reproduce the simulation results. Usage instructions are provided within the archive.
(ZIP)
